# Seasonal and spatial assessment of aquatic pollution in the Mediterranean crab *Callinectes sapidus*

**DOI:** 10.1038/s41598-026-61684-3

**Published:** 2026-07-21

**Authors:** Asmaa Ahmed El-Kenway, Hadeer Abd El-hak Rashed, Samya Hussein Mohammad, Hala E. Abd-Alaty

**Affiliations:** Zoology Department, Faculty of Science, Port Said University, Port Said, Egypt

**Keywords:** Water pollutants, *C. sapidus*, Oxidative stress biomarker, Parasitic stressor, Ecology, Ecology, Environmental sciences

## Abstract

Pollution in the Mediterranean Sea driven by heavy metal contamination, declining water quality, and rising parasite prevalence seriously threatens marine life and ecosystem health. This study examined the spatial and temporal variation of seawater pollution on the Mediterranean crab *Callinectes sapidus*. Seawater, sediment, and crab samples were seasonally collected from June 2023 to May 2024 at two sites (El Bogus, Site1, and El Matar, Site2) in Port Said, Egypt. Heavy metals analysis showed elevated levels in the sediments compared to those in the water and crab muscles. Fortunately, Cd was undetected. However, Pb showed higher concentrations compared to Cu. Its concentration was the highest at Site 2 in all seasons. The current study proved that Cu concentration was within the safe limits in the crab muscles. Whereas Pb exceeded the WHO limit, but was still within the FAO limit. MDA and comet assays revealed elevated oxidative stress. The recovered parasites collected from *C. sapidus* belonged to Ciliphora, Rotifera, and Nematodes. The total parasitic infection was higher at Site1 (62.8%) than at Site 2 (38.4%). Male crabs and those that were larger in size and greater in carapace length exhibited the highest infection dominance compared to females. This study highlighted the impact of spatial and temporal variations of seawater pollution on the Mediterranean crab *C. sapidus*, with respect to external (heavy metals) and internal (parasitic infection) stressors. Hence, regular monitoring and pollution control, especially targeting lead contamination and parasitic infections, are essential to protect the aquatic ecosystem and public health.

## Introduction

The Mediterranean Sea is a unique and diverse marine ecosystem that connects three continents, Asia, Africa, and Europe and supports thousands of species^1^. It plays a vital role in supporting Egypt’s fishery resources. It is located at the northern entrance of the Suez Canal. Port Said has access to rich marine biodiversity, making it an important source of livelihood for local fishermen^2^. Due to the nutrient-rich waters and favorable climate, the Mediterranean coast of Port Said remains one of the key areas for sustainable fish production in Egypt. Lake Manzala is the largest lake of Egypt that branched from the Mediterranean and is considered the most productive for fisheries. It is located in the north-eastern corner of the Nile delta. It is separated from the Mediterranean Sea by a sandbar, through which it is connected to the sea by a channel called bogus^3^.

There is a complex environmental assemblage of known xenobiotics and an increasing number of undiscovered contaminants that negatively impact aquatic ecosystems as well as human health^3,4^. Among Egypt’s environmental and public health issues is water pollution, which is a significant issue^5^. Pollutants resulting from many sources such as, industry, transportation, and domestic sewage, are now considered to be worldwide environmental contaminants that cannot be disregarded. This is because of their widespread occurrences and residues in the environment, particularly in marine matrices and the direct or indirect health risks they pose to people^6^. These metals are of interest because of their ability to bioaccumulate in organisms and potentially biomagnify through food chains, making them relevant indicators of ecosystem contamination. Studying them together also allows for the assessment of combined and interactive effects, providing a more realistic understanding of environmental exposure conditions^7^

Recently, The Mediterranean Sea has been of great concern because it is exposed to different pollutants^8,9^. In addition to oil pollution from ships, the Mediterranean Sea at Port Said is exposed to agricultural drains tainted with organic debris, fertilizers, pesticides, hazardous industrial wastes, and household sewage^10^. Therefore, Manzala Lake is susceptible to different sources of contamination^11,12^. All these pollutants are highly toxic to marine life and can threaten human and ecosystem health^13,14^.

Numerous studies have demonstrated that oxidative stress is exacerbated when organisms are exposed to environmental pollutants. Oxidative stress can change several biochemical, cellular, and physiological processes that could either directly or indirectly impact aquatic species’ ability to survive^15–17^. Reactive oxygen species (ROS) produced by many substances emitted from industrial processes, xenobiotics, and pesticides may exacerbate oxidative stress^18,19^. Exposure to heavy metals can cause DNA damage by generating reactive oxygen species, but it can also cause cellular alterations that can affect the competitive balance between repair pathways and change how the double-strand break repair process turns out^20^. Heavy metals cause a great threat to both aquatic organisms and the quality of water because of their high toxicity and bioaccumulation capability^7^.

Parasitic diseases can significantly burden aquatic populations due to their significant physiological and ecological host effects^21^. Parasitic infection can change the symmetry of competition between infected and uninfected individuals, alter host phenotypes, and adversely affect the fitness and population dynamics of hosts^22^. Communities of hosts and their parasites have intricate interactions; the traits of the hosts, parasites, and environment all influence one another. The environment may be a strong predictor of the diversity and abundance of parasites for a particular host species. Alterations in the host’s phenotype, including morphological, physiological, and immunological alterations, are frequently important markers of the existence and severity of specific parasite interactions since the host mediates a large number of within-host parasite interactions^23^.

Heavy metal contamination can increase host susceptibility to parasitic infection by suppressing immune responses, while parasites may accumulate metals at high concentrations and thus act as bioindicators of environmental pollution. Moreover, combined exposure to heavy metals and parasitic infection may have synergistic adverse effects on host physiology (Akinsanya et al., 2020).

Parasitic infections can cause an imbalance between the production of reactive oxygen species (ROS) and the body’s ability to counteract them using antioxidants. This condition, known as oxidative stress, can harm important cellular structures, especially DNA. Damaged DNA may appear as breaks in the strands, changes in bases, or even instability in chromosomes, which could affect cell function and increase the risk of mutations. Studying how parasites cause this damage helps researchers find ways to protect the health of infected hosts^24^.

The Atlantic coast of the Americas is endemic to the blue crab *Callinectes sapidus*^25,26^. Globally, since it has been brought to the Mediterranean Sea, the European North East Atlantic, Baltic Sea, Black Sea, North Sea, and Japan, it is among the most invasive marine species in the world^25,27^. Due to its euryhaline and eurythermal characteristics, *C. sapidus* may adapt to a variety of situations. *C. sapidus*'s great fecundity, broad ecological tolerance, and powerful swimming ability may enable it to colonize numerous seas^28^. The use of the invasive crab species as a bioindicator has been clarified in the revised manuscript. This species was selected due to its wide distribution in coastal habitats, and it is characterized by high capacity for bioaccumulation of heavy metals, which makes it a reliable indicator of environmental contamination and ecosystem health. In addition it is considered as one of the cheap and commercial food sources in the Portsaid governrate, so we need to study the different stressors represented by heavy metals as environmental stressors and parasites as biological stressors. and the degree of impacts on such edible food type Hence, The study hypothesized that spatial and seasonal variations in heavy metal contamination would significantly affect the health status of crabs, as reflected by oxidative stress biomarkers, DNA damage, and parasitic infection levels.

## Material and methods

### Study area

**Figure 1:**
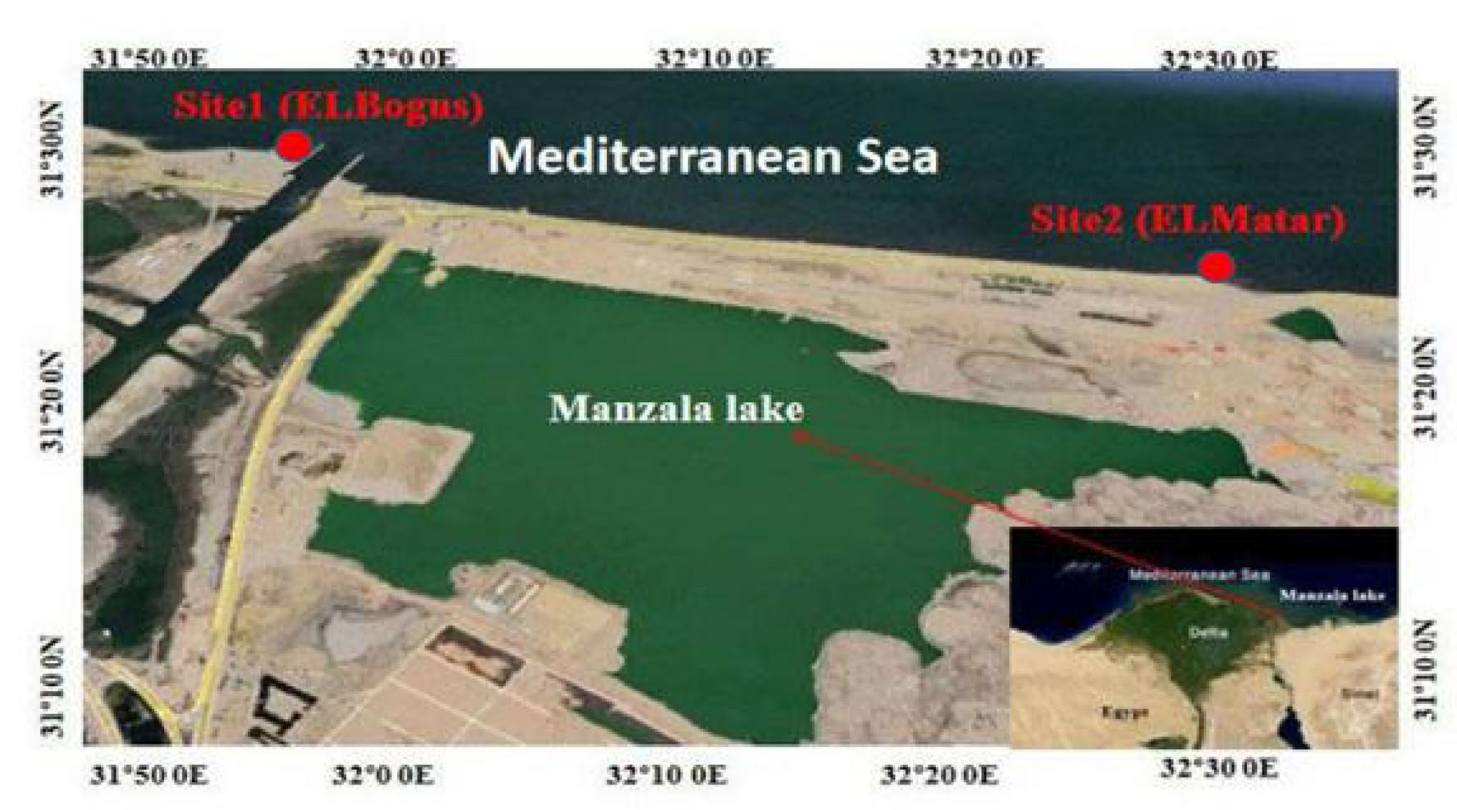
Map of the sampling sites, generated using Google Maps.

The samples were collected from two sites in the Port Said Governorate, Egypt. The first site, El Bogus, is an outlet situated along the northeastern side of Lake Manzala within the coastal sandbar, approximately 5 km west of Port Said on the Mediterranean Sea (Fig. 1). The second site, El Matar, is located at 31°30′ 0′ N and 32° 30′ 0′ E on the Mediterranean Sea.The sampling sites were selected to represent two ecologically important areas along the Mediterranean coast. El-Bugas is characterized by intense fishing activities and continuous water exchange and subjected to anthropogenic inputs, including fishing operations, boat traffic, and land-based sources of pollution,while El-Matar is influenced by maritime activities and coastal urbanization.

### Sampling of the specimens

Specimens of seawater, sediment, and *C. sapidus* were seasonally collected from June 2023 to May 2024. Crab samples were captured at a depth of 2 m using gill nets and rinsed with deionized distilled water to remove any surface debris. These specimens were transported in containers filled with the site water to maximize their survival time and were delivered directly to the laboratory of parasitology at the Faculty of Science, Port Said University, Egypt. Crab specimens (88 ± 2) were collected seasonally from each sampling site. Additionally, three sediment samples of water and sediment were separately collected from each site seasonally. Details regarding sample preservation have also been added, indicating that crab specimens were transported to the laboratory in insulated ice boxes and processed on the same day of collection.

Additionally, water samples were collected using glass bottles at the same depth (2 m) and water samples were filtered through a 0.45* Mm* membrane filter to remove suspended particles then acidified with concentrated nitric acid to pH below 2 and stored at 4℃ until heavy metal analysis. This procedure was performed to preserve the samples and ensure accurate determination of dissolved metal concentrations used in the calculation of BCF values then frozen until analysis. Sediment samples were obtained from the bottom of both sites using a Grab Sampler. The sediment samples were placed in clean plastic bags, transported to the laboratory, air-dried, sieved using a 2 mm mesh, and stored at − 20 °C until analysis^29^.

### Ethics approval and sample collection

All wild Callinectes sapidus used in this study were collected under appropriate permits issued by the Egyptian Environmental Affairs Agency (Research code. ERN: PSU. SCI.137. 2025).

### Morphometric indices

The samples were weighed using a Kinlee Electric Sensitive Balance with a precision of 0.001 g **(**model: ATZ520). The width and length of each crab were measured with a 0.05 mm vernier caliper. Sex determination was conducted prior to dissection, with male crabs identified by their narrow abdomens and females by their broader abdomens.

### Physico-chemical parameters

Physicochemical parameters, including pH, temperature, total dissolved solids (TDS), salinity, and conductivity, were directly measured in the field. pH levels were determined using a digital pH meter (MW Model 102). Water temperature was measured using a thermometer, while total dissolved solids and conductivity were assessed using a digital TDS and conductivity meter (YSI Model 33 S.C.T). Salinity was measured using a salinity refractometer designed for seawater and aquarium applications (RHS Model 10 ATC).

### Heavy metal determination

#### Preparation and digestion of samples

The standard reagents for primary heavy metals, acquired from Merck, Germany, possessed an exceptional purity of 99.98%. Ultra-pure nitric acid (HNO₃) was employed for sample digestion, while all other acids and chemical compounds, sourced from Merck (Germany) or Scharlau (Spain), were of high analytical grade.

A 100 mL water sample was combined with 10 mL of nitric acid (HNO₃) and subjected to heating on a hot plate at approximately 70 °C for 8 h. Following the cooling process, 4 mL of 30% hydrogen peroxide (H₂O₂) was introduced, and the mixture was reheated at the same temperature for an additional 4 h. The resulting solution was then diluted to a final volume of 25 mL using distilled water. This procedure was conducted in triplicate for each season to ensure accuracy and reproducibility^30^.

Sediment samples were oven-dried at 70 °C for 24 h. The following day, they were sieved using a 0.75 mm plastic mesh. 0.5 g of the sediment was then subjected to digestion in 10 mL of HNO₃ overnight. Subsequently, the samples were heated on a hot plate at 70 °C for 8 h. After cooling, 4 mL of 30% (H₂O₂) was added, and the mixture was reheated at the same temperature for an additional 4 h. Finally, the digested solution was diluted to a final volume of 25 mL using distilled water^31^.

The muscle tissues of the crabs were thoroughly cleaned and oven-dried at 65 °C until most of the moisture had evaporated. The temperature was then increased to 80 °C, and the samples were left to dry for 24 h. The dried tissues were finely ground into a powder. 0.25 g portion of the powdered tissue was digested in 5.0 mL of (HNO₃) by heating on a hot plate at 70 °C for 8 h. Once the solution had cooled, 2 mL of 30% hydrogen peroxide was added, and the mixture was reheated at 70 °C for an additional 4 h. Finally, the digested solution was diluted to a final volume of 25 mL using distilled water^30^.

#### Heavy metals evaluation

Quality Control (QC) and Quality Assurance (QA) procedures were implemented to ensure the reliability of heavy metal analysis, with QC samples showing recoveries within the certified range of 95–100% for the tested metals.. Three heavy metals (Cu, Cd, and Pb) were chosen to be evaluated in water, sediment, and muscle samples through a PerkinElmer flame atomic spectrometer (model: AS 2380, Chemical Merk, Germany). Analytical quality assurance was ensured using certified reference standard solution (PerkinElmer).

Strict protocols were followed during sample collection, storage, and preparation to prevent contamination. All equipment was thoroughly cleaned with acid-washed deionized water before use. Analytical instruments were calibrated using certified standard solutions of heavy metals, and calibration curves were established to ensure accuracy and precision in metal concentration measurements. Blank samples (free from heavy metals) and triplicate samples were analyzed alongside test samples to detect potential contamination and assess the consistency of the results.

To validate the analytical methods, recovery tests were conducted by spiking samples with known concentrations of heavy metals, and the percentage of recovered metals was compared to expected values. Instrument precision was evaluated by repeatedly analyzing standard solutions, while accuracy was confirmed by comparing results with certified reference materials. Data validation was meticulously reviewed, with any discrepancies investigated and rectified to ensure the integrity and reliability of the findings.

#### Biota sediment accumulation factor (BSAF)

BSAF for heavy metal concentration in crab muscle represents the ratio of heavy metal concentration in crab muscle to their concentration in the sediment. It is calculated using the formula provided by Adolfsson-Erici et al.^32^ as follows:

$$BSAF = C_{c} /C_{s}$$

where *C*_*c*_ is the heavy metal concentration in crab muscle, and *C*_*s*_ is the heavy metal concentration in the sediment.

#### Bioconcentration factor (BCF)

BCF for heavy metal levels in crab muscle represents the ratio of heavy metal concentration in the crab’s muscle to its concentration in the surrounding aquatic environment. It is calculated using the formula provided by Adolfsson-Erici et al.^32^ as follows:

$$BCF = C_{c} /C_{W}$$

where *C*_*c*_ is the heavy metal concentration in crab muscle, and *C*_*w*_ is the heavy metal concentration in the water.

### Determination of malondialdehyde (MDA) level in the muscles

MDA was colorimetrically estimated using the Ox Select™ TBARS Assay Kit (Cell Biolabs, Inc., Catalog no. STA_330, San Diego, CA 92,126) according to the manufacturer’s recommendations.

### Comet assay

The comet test was performed according to Singh et al*.* (1988). 0.5 g of crushed muscle was added to 1ml ice-cold phosphate buffer saline (PBS), then filtered, and 100 μL of cell suspension was mixed with 600 μL of low-melting agarose. On a pre-coated slide, the mixture was spread, followed by immersion of the slides in lysis buffer (0.045 M TBE, pH 8.4, containing 2.5% SDS) for 15 min. In the electrophoresis chamber, the slides were put and the conditions were 2v/cm for 2min. and 100mA. Slides were stained with 20 μg/ml ethidium bromide at 4°C. For each sample, DNA fragment migration patterns of 100 cells were evaluated using a fluorescent microscope. The lengths of comet tails were assessed from the nucleus center to the end of the tail. A comet image analysis software developed by Kinetic Imaging, Ltd. (Liverpool, UK) linked to a CCD camera was used to assess cellular DNA damage according to the length of DNA migration and the percentage of migrated DNA. 50 to 100 cells were randomly selected per sample.

### Parasitological examination

The crabs were placed ventral side up on a dissecting dish. The carapace was carefully removed using scissors to expose the internal organs. Muscle and gill tissues were excised and transferred separately into clean Petri dishes, where they were rinsed with a 0.9% NaCl saline solution to eliminate any residual tissue debris. For muscle examination, 0.1 g from each sample was compressed between two clean glass slides for microscopic analysis^33^. Similarly, 0.1 g of gill tissue was filtered using a filtration apparatus, and the supernatant was collected for further examination^34^. The isolated parasites were photographed using a digital camera (OptikaB5).

#### Parasitological identification

The parasites were classified based on differences in their shape and morphology. Ciliophora were identified by their general body appearance, size, external ciliary characteristics, and stalk morphology^35^. Rotifera were identified by the presence of a corona formed in the anterior region characterized by a cluster of fine, hair-like projections. Finally, the nematodes were classified based on the morphology, internal structures, particularly the germinative zone, and the posterior end. Additionally, egg morphology was utilized in the identification process.

#### Occurrence of the parasites

The prevalence, mean intensity, and abundance of recovered parasites were registered. Prevalence, or infection rate, is defined as the percentage of infected crabs relative to the total number of crabs examined. Intensity refers to the number of parasites present in a single infected crab, while abundance represents the total parasite burden, including both infected and uninfected specimens.

Parasite dominance was assessed based on seasonal variations and biological factors of the crabs, including length, weight, and sex. Crab samples were categorized into two length and weight classes at each site. At Site 1, crabs were classified into two groups: class I (<56 cm and <110g), class II (>56cm and >110g). Similarly, at Site 2, the groups were class I (<49.6 cm and <76.6 g) and class II (>49.6 cm and >76.6 g).

### Statistical analysis

The analysis was carried out by mean of SPSS version 20. The results were expressed as mean ± standard deviation. The assumptions of normality and homogeneity of variances were tested before Two-way ANOVA. Tukey’s HSD post hoc test was conducted whenever significant differences were detected was considered statistically significant when p < 0.05. Pearson correlation analysis was applied to correlate physicochemical parameters and heavy metals with MDA, tail moment, and parasite prevalence. In addition, Origin software was employed to generate a heat map to visualize the correlation matrix.

## Results

### Physicochemical parameters

At Site 1, temperature peaked during summer (28.6 ± 0.28 °C) and declined notably by winter (19.0 ± 1.0 °C). A similar seasonal trend was observed in pH, which recorded a summer maximum of 8.56 ± 0.42, before dipping to 7.56 ± 0.21 in winter. Salinity values fluctuated slightly, reaching 33.66 ± 1.52 PSU in winter, while maintaining relatively the lowest levels in spring (30.0 PSU). The electrical conductivity remained fairly stable across the year, though a mild increase was noted in summer (105.40 ± 6.47µS/cm). Meanwhile, TDS concentrations were more variable, with a notable accumulation in summer (82.6 ± 15.5 mg/L) and a marked reduction during autumn (61.06 ± 0.90 mg/L) (Table 1).

**Table 1:** Seasonal variations in physicochemical parameters of seawater in the present study.

Site	Parameter	Temperature (°C)	pH	Salinity (PSU)	Conductivity (µS/cm)	TDS (mg/L)
	Season					
Site1	Summer	28.6 ± 0.28	8.56 ± 0.42	33.0 ± 1.73	105.40 ± 6.47	82.6 ± 15.5
	Autumn	23.6 ± 1.53	8.17 ± 0.37	31.3 ± 0.57	101.13 ± 1.41	61.06 ± 0.90
	Winter	19.0 ± 1.0	7.56 ± 0.21	33.66 ± 1.52	102.56 ± 2.60	62.46 ± 1.67
	Spring	23.0 ± 3.61	8.00	30.0	102.8 ± 1.92	62.0 ± 2.6
Site2	Summer	28.7 ± 0.46	5.33 ± .0.32	38.0 ± 1.0	309.3 ± 20.64	153.66 ± 47.0
	Autumn	24.6 ± 1.5	5.93 ± 0.38	37.3 ± 0.57	370.66 ± 60.05	200.3 ± 22.8
	Winter	18.6 ± 2.08	6.13 ± 0.23	38.6 ± 0.57	413.0 ± 31.57	213.66 ± 13.1
	Spring	23.66 ± 3.79	6.86 ± 1.03	38.3 ± 1.52	401.0 ± 1.0	200.0 ± 2.00

Data resented as Mean ± SD. pH, power of hydrogen and TDS, Total dissolved salts.

At Site 2, temperature followed the same seasonal trend, with summer being the warmest (28.7 ± 0.46 °C) and winter considerably cooler (18.6 ± 2.08 °C). pH values, however, were consistently lower than those at Site 1, fluctuating between 5.33 ± 0.32 in summer and 6.86 ± 1.03 in spring. Notably, salinity remained high and steady across all seasons, averaging around 38 PSU. Conductivity, on the other hand, escalated during winter, reaching 413.0 ± 1.57 µS/cm, while dropping to 309.3 ± 20.64 µS/cm in summer. TDS levels mirrored this pattern, with elevated readings in winter (213.66 ± 13.1 mg/L) and a noticeable decline during the summer months (153.66 ± 47.0 mg/L) (Table 1).

### Heavy metals

#### Heavy metal concentrations in water samples

The concentration of heavy metals was recorded in water samples at both sites (Table 2). The seasonal distribution of Cu concentrations at Site 1 followed the order: spring and winter > autumn and summer, whereas at Site 2, the order was slightly different (spring > winter > autumn and summer). Similarly, Pb concentration at Site 1 followed the order: spring > winter > autumn and summer, while at Site 2, it was recorded as spring > winter > summer > autumn. Furthermore, Cd was not detected in any of the examined samples. However, there was a significant difference of the concentration of Cu between seasons as well as between sites (*p* = 0.007 and 0.01) for them respectively, and the concentration of Pb had a highly significant difference in the four seasons (*p* < 0.001) but not a significant difference between two sites (*p* = 0.15).

**Table 2:** Heavy metal concentrations in the seawater, sediment and crab muscles from both sites at different seasons.

	Metal	Cd				Pb				Cu		
	Season	Water mg/L		Sediment mg/kg dry weight	Muscles mg/kg wet weight	Water mg/L		Sediment mg/kg dry weight	Muscles mg/kg wet weight	Water mg/L	Sediment mg/kg dry weight	Muscles mg/kg wet weight
Site 1	Summer	< LOD		0.11 ± 0.00	0.41 ± 0.17	0.02 ± 0. 01		1.2 ± 0.03	0.95	< LOD	< LOD	< LOD
	Autumn	< LOD		0.22 ± 0.03	0.53 ± 0.01	0.02 ± 0.00		1.53 ± 0.01	0.82	< LOD	< LOD	< LOD
	Winter	0.02 ± 0.00		1.7 ± 0.003	1.06 ± 0.03	0.05 ± 0.00		1.3 ± 0.05	1.02 ± 0.01	< LOD	< LOD	< LOD
	Spring	0.02 ± 0.00		2.62 ± 0.01	1.97 ± 0.03	0.06 ± 0.00		3.02 ± 0.01	2.77 ± 0.01	< LOD	< LOD	< LOD
Site 2	Summer	< LOD		0.11 ± 0.03	0.38 ± 0.16	0.03 ± 0.01		1.68 ± 0.07	1.67 ± 0.07	< LOD	< LOD	< LOD
	Autumn	< LOD		0.19 ± 0.04	0.47 ± 0.04	0.02 ± 0.01		2.2 ± 0.03	1.29 ± 0.04	< LOD	< LOD	< LOD
	Winter	0.19 ± 0.03		2.87 ± 0.03	3.10 ± 0.03	0.06 ± 0.01		1.95 ± 0.04	1.60 ± 0.04	< LOD	< LOD	< LOD
	Spring	0.2 ± 0.02		4.0 ± 0.01	3.11 ± 0.05	0.09 ± 0.02		3.27 ± 0.01	3.37 ± 0.03	< LOD	< LOD	< LOD

Data presented as Mean ± SD.

< LOD = Below the limit of Detection.

#### Heavy metal concentrations in the sediment samples

The concentrations of heavy metals in the sediment from both sites, considering seasonal fluctuations, are documented in Table 2. Cu followed the same order at both sites: spring > winter > autumn > summer. Similarly, Pb exhibited a consistent pattern across both sites but in a different arrangement: spring > autumn > winter > summer. Cd was not also detected in the sediment samples. There was a significant difference across seasons for Cu *p* < 0.001 but no significant was found between sites (*p* = 0.30). For Pb concentration, as with Cu, exhibited a significant difference within four seasons (*p* < 0.001) but not between sites (*p* = 0.09).

#### Heavy metal concentrations in the crab muscle samples

The concentrations of heavy metals in the crab are displayed in Table 2. At Site 1, Pb had the highest concentration, with the following decreasing concentrations: spring > winter > summer > autumn. At Site 2, Pb had a higher concentration than in site 1, with a different arrangement: spring > summer > winter > autumn. Cu, on the other hand, had the same order at the two sites: spring > winter > autumn > summer. Finally, Cd was not detected in the two sites. There was a highly significant difference (*p* < 0.001) for Cu and Pb concentration between seasons, but the sites exhibited no significant difference (*p* = 0.09).

#### Biota sediment accumulation factor (BSAF) of heavy metals levels in *C. sapadius* muscle tissue

At Site 1, Pb concentrations were generally higher than Cu across all seasons, except in spring. BSAF for Pb followed a decline order: winter > summer, autumn, and spring. At the same site, Cu was undetectable in summer and autumn, while its concentration in spring exceeded that in winter (Table 3).

**Table 3:** Biota-Sediment Accumulation Factor (BSAF) and Bioconcentration Factor (BCF) of three heavy metals (Cu, Cd, and Pb) in the present study.

	Metal	BSAF			BCF		
	Season	Cu	Cd	P b	Cu	Cd	P b
Site 1	Summer	0	0	0.01 ± 0.05^a^	5.18 ± 0.09^a^	0	0.32 ± 0.42^a^
	Autumn	0	0	0.01 ± 0.03^a^	2.46 ± 0.05^a^	0	0.53 ± 0.03^a^
	Winter	0.01 ± 0.02^a^	0	0.03 ± 0.02^a^	0.62 ± 0.03^a^	0	0.76 ± 0.06^a^
	Spring	0.07 ± 0.01^a^	0	0.01 ± 0.00^a^	0.75 ± 0.02^a^	0	0.92 ± 0.01^a^
Site 2	Summer	0	0	0.02 ± 0.01^b^	5.01 ± 0.16^b^	0	0.94 ± 0.08^b^
	Autumn	0	0	0.01 ± 0.02^b^	2.39 ± 0.07^b^	0	0.60 ± 0.02^b^
	Winter	0.07 ± 0.01^b^	0	0.03 ± 0.01^b^	1.09 ± 0.02^b^	0	0.81 ± 0.03^b^
	Spring	0.05 ± 0.28^b^	0	0.03 ± 0.02^b^	0.77 ± 0.02^b^	0	1.03 ± 0.01^b^

Data represented as Mean ± SD. Values with different superscripts in each season at the different sites indicate significant difference at p < 0.05.

At Site 2, Pb and Cu exhibited the highest BSAF values in winter and spring. Cu was entirely absent in the remaining seasons. BSAF concentration of Pb followed the order: winter and spring > summer > autumn (Table 3).

#### Bioconcentration factor (BCF) of heavy metals levels in *C. sapadius* muscle tissue

At Site 1, the BCF of Cu was higher than that of Pb in three seasons, but not in winter. The BCF of Cu followed the order: summer > autumn > spring > winter, whereas Pb exhibited a different trend: spring > winter > autumn > summer. At Site 2, Cu also had a higher BCF than Pb in three seasons, except in spring. The BCF of Cu was highest in summer, followed by autumn, winter, and spring. In contrast, the BCF of Pb followed the order: spring > summer > winter > autumn (Table 3).

### MDA in *C. sapidus* muscle tissues

**Figure 2:**
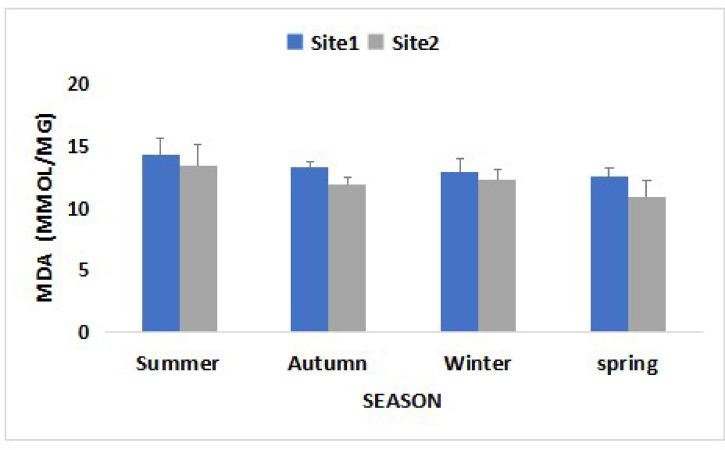
MDA levels in C*. sapidus* muscles during the four seasons at both sites.

The levels of MDA in C*. sapadius* muscle tissues at both sites during the four seasons are displayed in Fig. 2. The highest levels were recorded during summer, while the lowest were found in the spring. Additionally, levels of MDA increased in the crab muscle tissues collected from Site1 compared with those from Site2 in each season. However, this increase was not statistically significant (*p* > 0.05).

### Comet assay in* C. sapidus* muscle tissues

**Figure 3:**
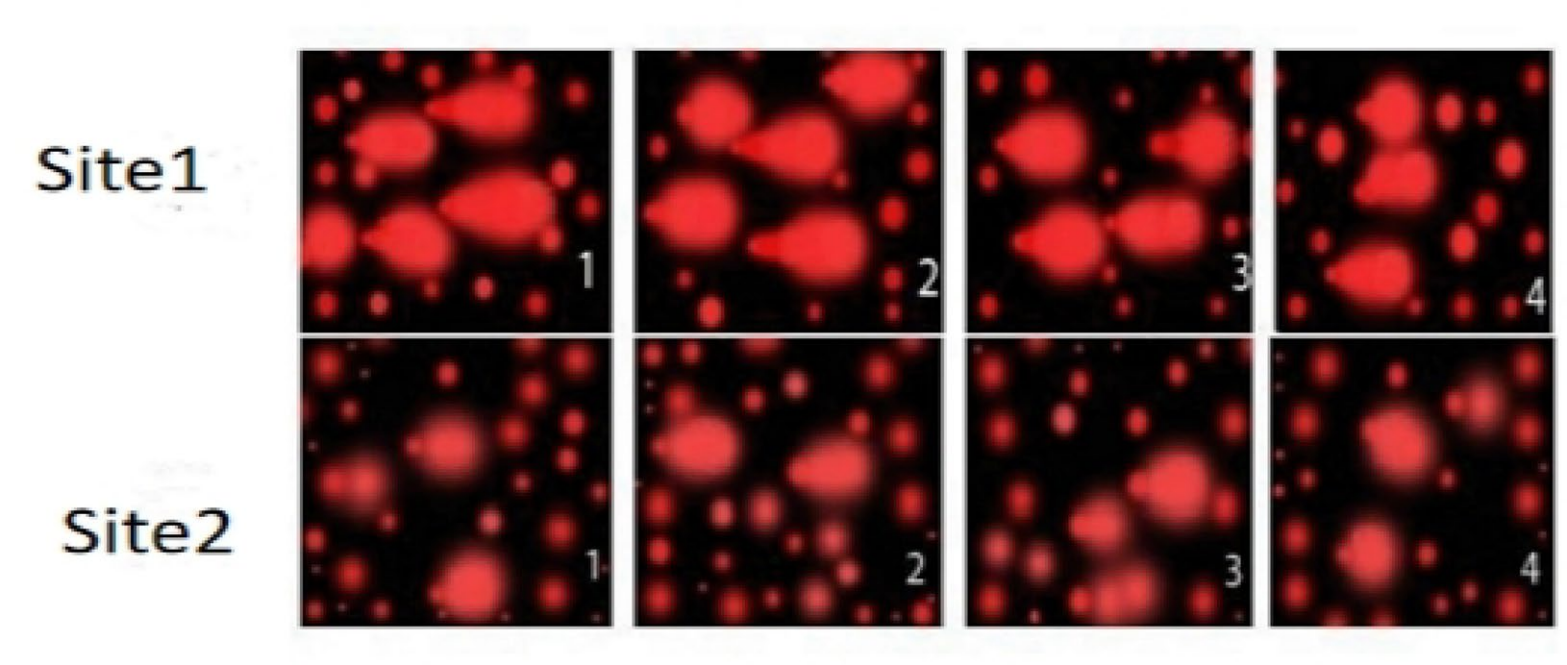
DNA in *C. sapidus* muscle collected from both sites at different seasons.** (1)** summer. **(2)** autumn. **(3)** winter. **(4)** spring.

The highest level of DNA damage was in spring and the lowest in autumn at Site1. While, the highest was in winter and the lowest in spring at Site 2. Also, the percentage of tail DNA, tail length, and tail moment are shown in Table 4, and Fig. 3. It is clear that DNA tail length in the crab muscle cells increased significantly during summer at Site1 with the values of (2.66 ± 0.101) compared to that at Site2.

**Table 4:** Levels of DNA damage in the muscle of *C. sapidus.*

	Season	Tail length Mm	Tail DNA%	Tail moment
	Site			
Site1	Summer	2.66 ± 0.101^a^	2.55 ± 0.058	6.79 ± 0.406
	Autumn	2.08 ± 0.109	2.29 ± 0.237	4.44 ± 0.65
	Winter	3.02 ± 0.080	3.02 ± 0.147	9.1 ± 0.33
	Spring	3.63 ± 0.399^a^	4.02 ± 0.292^a^	14.7 ± 5.7^a^
Site2	Summer	2.21 ± 0.15^b^	2.42 ± 0.085	5.34 ± 0.55
	Autumn	2.00 ± 0.160	2.02 ± 0.121	3.99 ± 0.62
	Winter	2.77 ± 0.096	2.83 ± 0.085	7.87 ± 0.21
	Spring	2.21 ± 0.174^b^	2.54 ± 0.281^b^	2.55 ± 1.04 ^b^

Data are presented as Mean ± SD. Values with different superscripts in each season at the different sites indicate significant difference at *p* < 0.05.

The percentage of tail DNA 2.55 ± 0.058 and tail moment 6.79 ± 0.406 in crab muscle at Site1, where higher than those at Site 2 (2.42 ± 0.0851) and (5.34 ± 0.55) for them, respectively. However, this increase wasn’t significant (*p* = 0.1). On the other hand, there was a nonsignificant increase in DNA damage during either winter or autumn at Site1 comparing with Site2. Moreover, the level of DNA damage in the crab muscle cells during spring was significantly (*p* = 0.02) higher at Site1 with DNA% 4.02 ± 0.292, tail length: 3.63 ± 0.399, and tail moment: 14.7 ± 5.7 compared with that at Site2 (DNA %: 2.54 ± 0.28, tail length: 2.21 ± 0.174, and tail moment: 2.55 ± 1.04).

### Parasitic identification

Out of 710 examined *C. sapidus*, different parasitic organisms were isolated from the infected muscle and gills. These organisms were ciliphorans with different species:* Mesanophrys s*p., *Pleuronema* sp., *Trichodina* sp*., Zoothamnium* sp., and *Vortcella s*p. There were also two distinct species of Rotirefra *Lecane* sp. and *Brachionus plicatilis.* Finally, the nematodes were represented by *Gammarinema* sp. (egg, Larvae and adults of both sexes).

#### Ciliphorans

##### ***Mesanophrys*** sp. (Family: Orchitophryidae)

**Figure 4:**
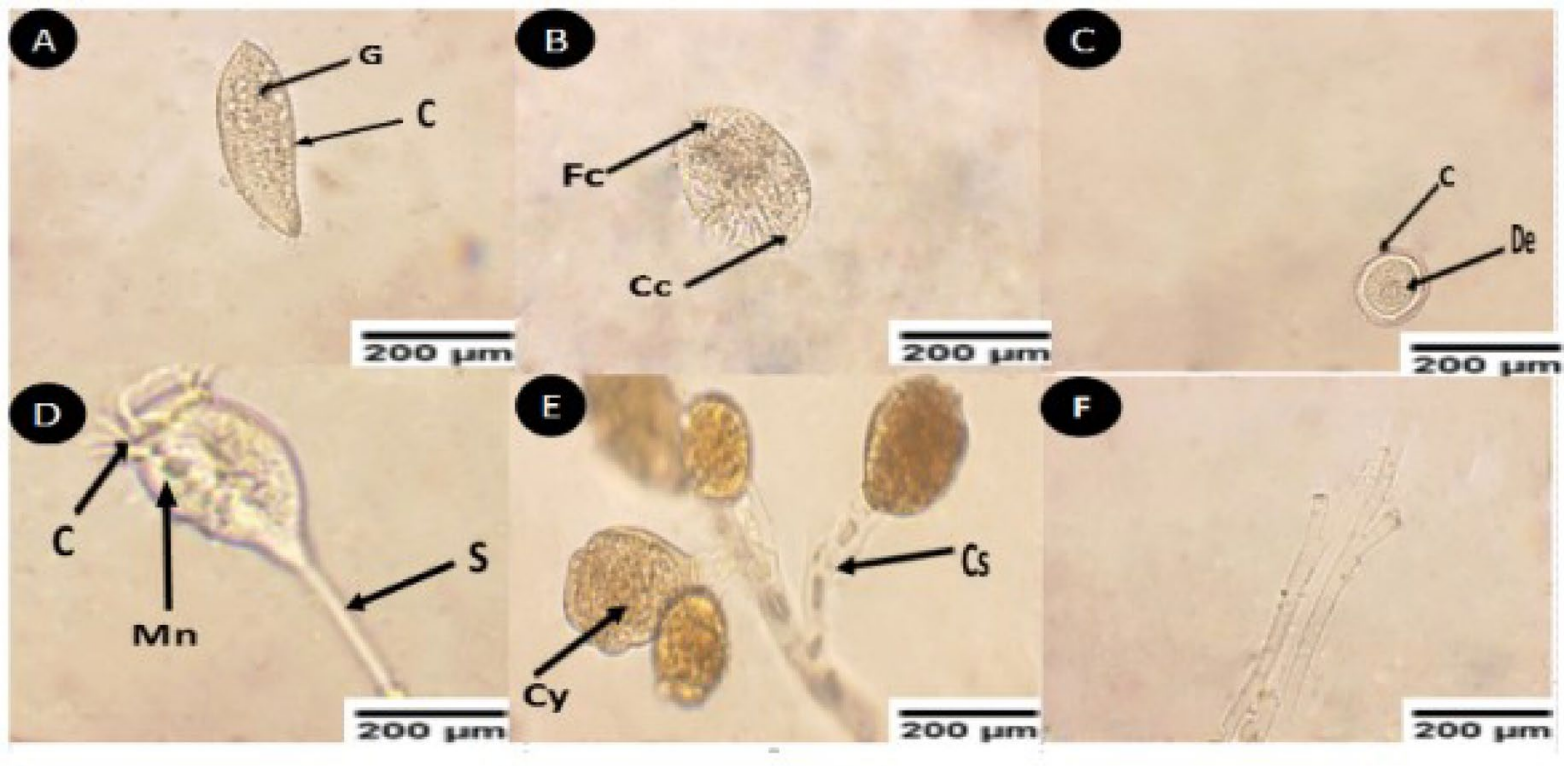
Ciliphora isolated from *C. sapidus* **(A)** *Mesanophrys* sp. **(B)** *Pleuronema* sp. **(C)** *Trichodina* sp.** (D)*** Vorticella* sp. **(E)*** Zoothamnium* sp. **(F)** *Zoothamnium* sp. stalk without head. C; cilia, G; granules, Fc; frontal cirri, Cc; caudal cirri, De; denticle, Mn; Macronucleus, S; Stalk, Cs; contractile stalk, Cy; cytoplasm. (magnification = 40x, scalebar = 200 µm).

The body was flattened, leaf-like, and oval in outline. It lacked a mouth and contractile vacuoles. The anterior end was semi-rounded, while the posterior end was pointed. The entire outer surface was covered with cilia. Several small, spherical, and uniformly sized granules were present (Fig. 4A).

##### ***Pleuronema*** sp. (Family: Pleuronematidae)

It exhibited an oval appearance; two types of cirri were present: frontal cirri at the anterior end and caudal cirri at the posterior end (Fig. 4B).

##### ***Trichodina*** sp. (Family: Trichodinidae)

Small, circular ciliates exhibited a disc-shaped appearance. A spiral arrangement of cilia, called the ciliary rings, that was centered around a structure referred to as the denticle (Fig. 4C).

##### ***Vorticella*** sp. (Family: Vorticellidae)

*Vorticella* sp. is found in solitary form, and it has a bell-shaped or inverted bell-shaped body. The body is attached to a long, contractile stalk that coils like a spring when disturbed. Around the edge of the bell, it has a ring of cilia (tiny hair-like structures). Internally, the macronucleus can be easily observed near the apical end (Fig. 4D).

##### ***Zoothamnium*** sp. (Family: Zoothamniidae)

This type of ciliate forms colonies. The organism’s body consisted of two distinct regions: a ciliated cup-shaped head and a robust stalk capable of morphological changinges, which contracted into a coiled structure upon disturbance. The head contained a dark brown cytoplasm (Fig. 4E). Notably, some individuals were observed lacking the head region (Fig. 4F).

#### Rotifera

##### ***Lecane*** sp. (Family: Lecanidae)

**Figure 5:**
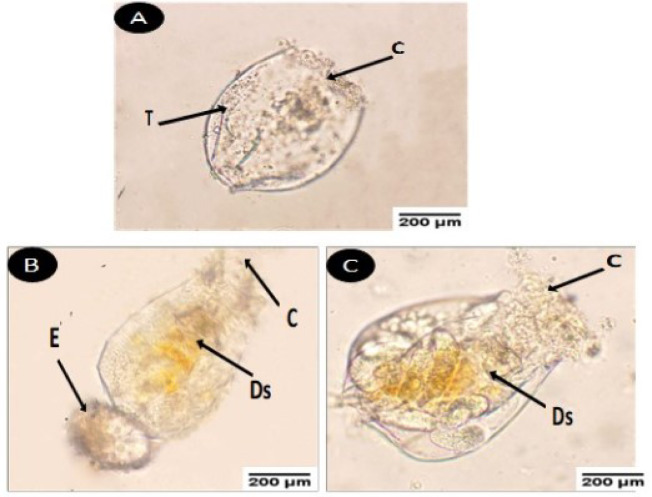
Rotirefra isolated from *C. sapidus*. **(A)*** Lecane* sp. (magnification = 40x, scale bar = 200 µm). **(B&C)** Female and male of *Brachionus plicatilis*, respectively (magnification = 40x, scale bar = 200 µm). C; corona, T; toe, Ds; Digestive system, E;Egg.

They were characterized by their floral, cup-shaped bodies. A cluster of fine, hair-like projections was present in the anterior region, forming the corona (Fig. 5A). Internally, a darkened area was observed, representing the digestive system. In the posterior region, the anchoring foot was present, with a terminal toe in a retracted position within the body.

##### ***Brachionus plicatilis*** (Family: branchinonidae)

Its body exhibited a cup-shaped appearance with structural similarities to *Lecane* sp. (Fig. 5B,C). The corona, with its cilia, was located anteriorly, while the digestive system displayed a yellow coloration. The foot could be either retracted within the body or extended externally. Females were easily distinguishable from males by the presence of an oval, brownish egg in the posterior region.

#### Nematode

##### ***Gammarinema*** sp. (Family: Monhysteridae)

**Figure 6:**
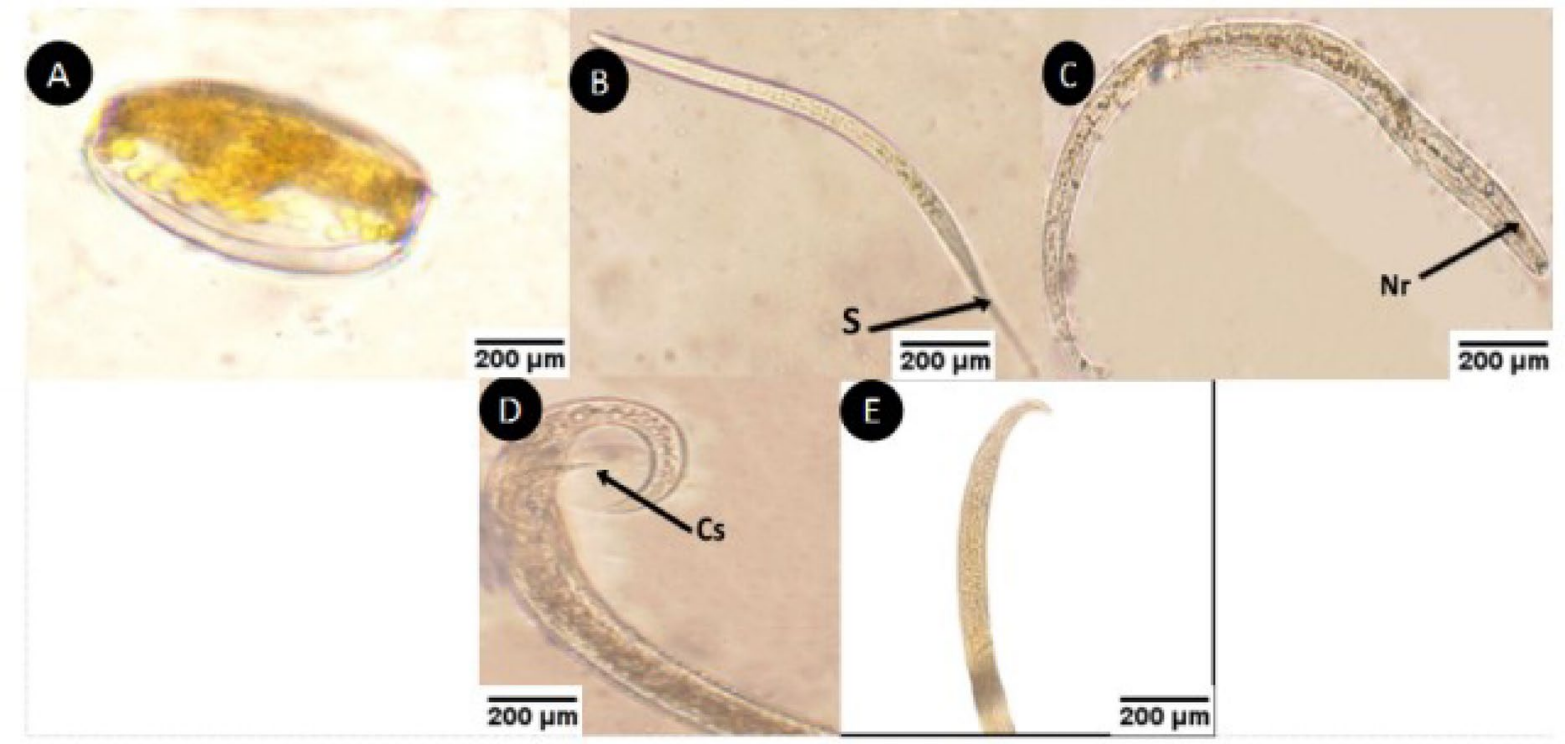
Whole mount of different developmental stages of *Gammarinema* sp. isolated from C. *sapidus*. **(A)** Egg of *Gammarinema* sp. **(B**) Larva of *Gammarinema* sp. **(C**) Adult of* Gammarinema* sp. **(D)** Posterior end of adult male **(E)** Posterior end of adult female. S; sheath, Cs; copulatory spicules, Nr; nerve ring. (magnification = 40x, scale bar = 200 µm).

Three developmental stages, egg, larva, and adult, were isolated from the crabs. The eggs were oval, with thin, double walls and yellow germinative contents. The larvae were thread-like, with a very thin posterior end known as the spicule sheath (Fig. 6B). Adult worms were cylindrical in shape (Fig. 6C). The posterior end was used to determine the sex of the worm. This region was more curved in males and characterized by the presence of copulatory spicules (Fig. 6D,E).

Crabs collected from Site1 exhibited a higher infection rate compared to those collected from Site2, with overall prevalence rates of 62.8% and 38.4%, respectively. Furthermore, both the abundance (4.18 ± 8.57) and intensity (22.18 ± 8.64) of infection were greater in the bogus group. Among the parasites identified, *Zoothamnium* sp. showed the highest infection rates in the muscles and gills; its total prevalence was 22.5% and 17.6% in Site1, and Site2 respectively (Table 5).

**Table 5:** Dominance of different parasites in the blue crab *C.* sapidus at both sites.

	Site1 (N = 339)				Site2 (N = 371)		
	Parasites	Gills	Muscles	Total	Gills	Muscles	Total
Prevalence (%)	*Mesanophrys sp.*	1.2	0.5	1.7	1	0.2	1.2
	*Pleuronema sp.*	2.1	1	3.1	0.3	0.5	0.8
	*Trichodina sp.*	4.8	1.5	5.2	2.4	1.3	3.8
	*Vorticella sp.*	3	1.5	4.5	0.1	0.2	0.3
	*Zoothamnium sp.*	12	10.5	22.5	8	10.66	17.6
	*Lecane sp.*	1.5	1.2	2.7	1.1	0.9	2
	*Brachionus plicatilis*	2.1	1.2	3.3	0.3	0.2	0.5
	*Gammarinea sp. (larva)*	5.3	2.4	7.4	1.5	1.1	3.8
	*Gammarinea sp. (adult)*	3.8	2.9	6.5	1.6	0.3	1.8
	Other	7.7	4.7	12.1	3.8	2.2	5.4
	Total	43.5	25.4	62.8	23.1	17.56	38.4
Abundance	Parasites	Gills	Muscles	Total	Gills	Muscles	Total
	*Mesanophrys sp.*	0.04 ± 0.21	0.09 ± 0.29^a^	0.13 ± 0.51	0.01 ± 0.08	0.04 ± 0.19^b^	0.05 ± 0.21
	*Pleuronema sp.*	0.03 ± 0.18	0.03 ± 0.18^a^	0.06 ± 0.36	0.036 ± 0.75	0.13 ± 0.33^b^	0.166 ± 1.08
	*Trichodina sp.*	0.11 ± 0.49	0.03 ± 0.0^a^	0.14 ± 0.63	0.03 ± 0.25	0.02 ± 0.17^b^	0.05 ± 0.30
	*Vortecella sp.*	0.28 ± 0.56	0.58 ± 0.88	0.86 ± 1.44	0.01 ± 0.77	0.01 ± 0.19	0.02 ± 0.97
	*Zoothamnium sp.*	1.45 ± 2.68	1.08 ± 2.24^a^	2.53 ± 3.36	0.19 ± 0.67	0.08 ± 0.33^b^	0.27 ± 0.83
	*Lecane sp.*	0.01 ± 0.08	0.02 ± 0.22	0.03 ± 0.29	0.01 ± 0.12	0.01 ± 0.11	0.02 ± 0.23
	*Brachionus plicatilis*	0.01 ± 0.11	0.01 ± 0.13	0.02 ± 0.24	0.01 ± 0.11	0.02 ± 0.13	0.03 ± 0.14
	*Gammarinea sp. (larva)*	0.07 ± 0.34^a^	0.02 ± 0.15^a^	0.09 ± 0.37	0.05 ± 0.27^b^	0.01 ± 0.10^b^	0.06 ± 0.33
	*Gammarinea sp. (adult)*	0.07 ± 0.39	0.03 ± 0.17^a^	0.10 ± 0.43	0.00 ± 0.16	0.02 ± 0.05^b^	0.02 ± 0.18
	Other	0.17 ± 0.87	0.05 ± 0.25	0.22 ± 0.95	0.02 ± 0.17	0.02 ± 0.17	0.05 ± 0.24
	Total	2.24 ± 5.89	1.94 ± 4.53	4.18 ± 8.57	0.186 ± 3.53	0.36 ± 2.13	0.736 ± 4.35
Intensity	Parasites	Gills	Muscles	Total	Gills	Muscles	Total
	*Mesanophrys sp.*	1.08 ± 0.89	1.03 ± 0.18^a^	2.11 ± 1.07	3.00 (n = 1)	1.00^b^ (n = 1)	4 (n = 1)
	*Pleuronema sp.*	1.25 ± 0.44^a^	1.87 ± 0.37	3.12 ± 0.72	1.02 ± 0.1	1.84 ± 0.24	2.94 ± 0.37
	*Trichodina sp.*	1.61 ± 1.08	1.80 ± 1.30^a^	1.70 ± 1.54	1.33 ± 1.00	1.40 ± 0.55^b^	1.36 ± 0.23
	*Vortecella sp.*	1.13 ± 0.15^a^	1.01 ± 0.2	2.13 ± 0.35	1.00^b^ (n = 1)	1.00 (n = 1)	2 (n = 1)
	*Zoothamnium sp.*	1.63 ± 0.16^a^	1.27 ± 0.81	2.9 ± 0.98	1.76 ± 1.14^b^	1.33 ± 0.48	2.22 ± 1.19
	*Lecane sp.*	1.01 ± 0.21	1.60 ± 0.78^a^	2.6 ± 0.89	2.50 ± 0.41	1.02 ± 0.22^b^	3.5 ± 0.71
	*Brachionus plicatilis*	10^a^ (n = 1)	1.00^a^ (n = 1)	11.00 (n = 1)	1.00 (n = 1)	1.00^a^ (n = 1)	2 (n = 1)
	*Gammarinea sp. (larva)*	1.02 ± 0.42	0.2 ± 0.14^a^	1.32 ± 0.56	1.31 ± 0.63	0.2 ± 0.19	1.50 ± 0.48
	*Gammarinea sp. (adult)*	0.7 ± 1.01	0.8 ± 0.10	1.50 ± 0.86	1.17 ± 0.17	0.2 ± 0.42	1.33 ± 0.58
	Other	1.85 ± 0.19	1.13 ± 0.34	2.98 ± 0.54	1.57 ± 1.16	1.13 ± 0.35	1.50 ± 1.00
	Total	10.23 ± 4.74	12.7 ± 3.90	22.18 ± 8.64	11.89 ± 4.79	11.7 ± 1.76	19.75 ± 4.15

Data are represented as Mean ± SD. Values with different superscripts in each season at the different sites indicate significant difference at p < 0.05. N; number of examined crabs, n; number of infected crabs.

**Figure 7:**
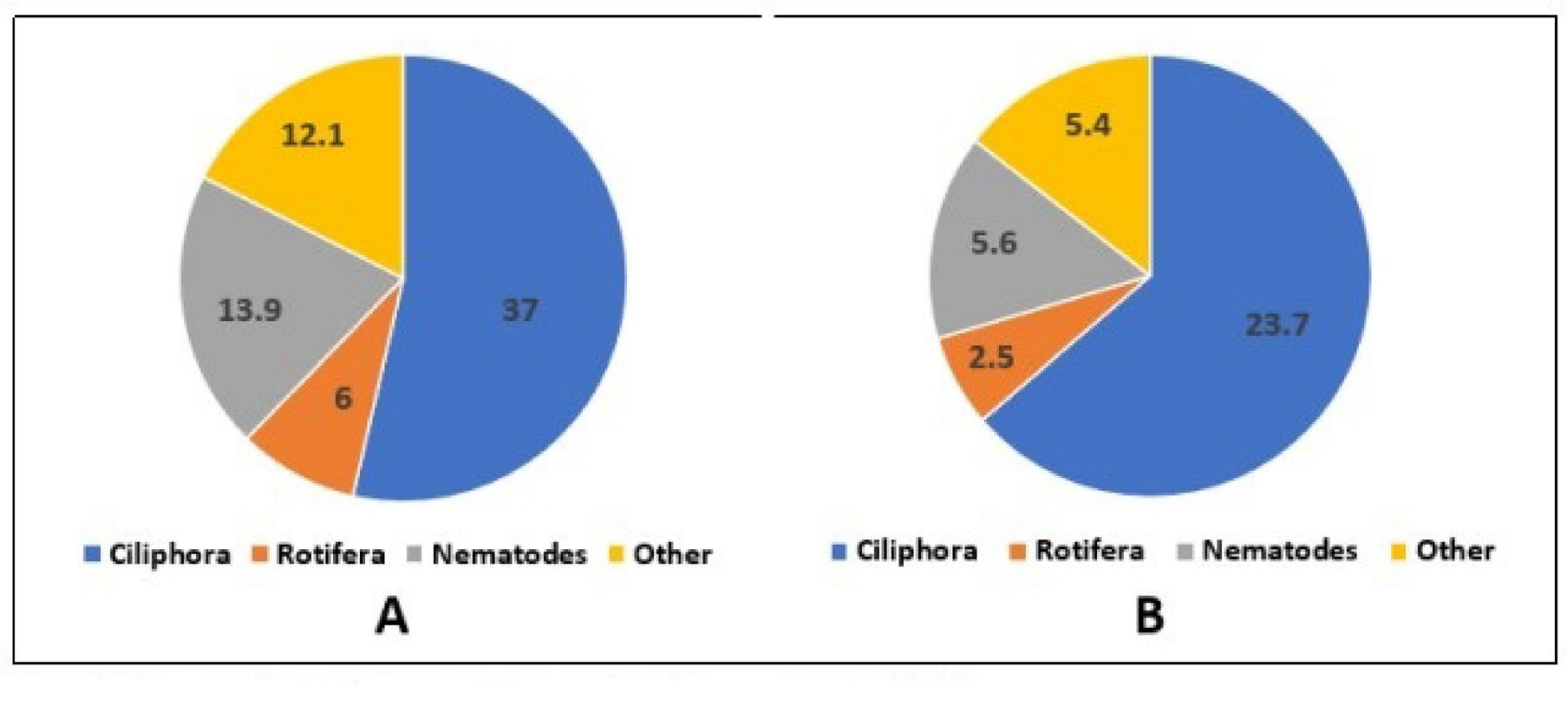
The infection rate between two sites. **(A)** Site1. **(B)** Site2.

The proportions of the parasite groups found in the sample within two sites. Ciliophora appears as the most common group, while Rotifera and Nematoda have much smaller percentages. The “Other” group represents a few less frequent types (Fig. 7).

The seasonal sequence of infection rates, arranged from highest to lowest, differed between the two sites. At Site1, the order was summer (51.3%), spring (22%), winter (20.81%), and autumn (18.64%). At Site2, the sequence was summer (38.5%), autumn (31.2%), spring (13.95%), and winter (3.50%). The remaining parasitological indices of the various parasites across seasons are presented in Table 6.

**Table 6:** Dominance of different parasites in the blue crab *C. sapidus* at different seasons.

	Site 1					Site 2			
	Parasites	Summer N = 103	Autumn N = 77	Winter N = 75	Spring N = 84	Summer N = 131	Autumn N = 90	Winter N = 75	Spring N = 75
Prevalence (%)	*Mesanophrys sp.*	1	1.2	0	1.5	2.1	0.1	0.1	0.2
	*Pleuronema sp.*	1.2	2.8	2.6	5.2	3.4	0.1	0.1	1
	*Trichodina sp.*	8.72	1.8	1.03	6.16	2.25	1.8	0	0
	*Vortecella sp.*	0.5	1.2	0	0	4.0	2.4	0.2	0.1
	*Zoothamnium sp.*	9.34	1.04	3.75	3.5	4.9	17.1	0.75	8.25
	*Lecane sp.*	1	1.1	3.5	0	3.5	0.6	0.9	0.9
	*Brachionus plicatilis*	0.2	2.8	3.6	0	5.6	0.1	1	0.5
	*Gammarinea sp. (larva)*	9.79	4.26	3.09	6.18	7.25	2.7	0	1.5
	*Gammarinea sp. (adult)*	1.31	5.04	1.03	3.85	5.5	0.9	0	0.75
	*Other*	16.03	1.4	2.21	3.85	1.5	5.4	0	0.75
	*Total*	51.3	18.64	20.81	22	38.5	31.2	3.50	13.95
Abundance	Parasites	Summer	Autumn	Winter	Spring	Summer	Autumn	Winter	Spring
	*Mesanophrys* sp.	0.04 ± 0.24^a^	0.42 ± 0.49^a^	0	0.06 ± 0.29^a^	0.01^b^ (n = 1)	0.02 ± 0.15^b^	0	0.30 ± 0.47^b^
	*Pleuronema* sp.	0.10 ± 0.41^a^	0.86 ± 0.35^a^	0.64 ± 0.93	1.98 ± 0.22^a^	0.02 ± 0.12^b^	0.38 ± 0.49^b^	1.25 ± 0.97	0.84 ± 0.99^b^
	*Trichodina* sp.	0.26 ± 1.01	0.08 ± 0.27	0.04 ± 0.35	0.12 ± 0.4	0.11 ± 0.36	0.06 ± 0.43	0	0
	vortecellasp.	0.05 ± 0.22	0.06 ± 0.41	0	0	0.04 ± 0.19	0.06 ± 0.38	0	0
	*Zoothamnium sp.*	3.24 ± 0.32^a^	1.95 ± 0.24^a^	1.80 ± 1.97^a^	4.82 ± 4.37^a^	0.25 ± 0.84^b^	0.46 ± 1.06^b^	0.03 ± 0.23^b^	0.33 ± 0.86^b^
	*Lecane* sp.	0.05 ± 0.25	0.04 ± 0.19	0.05 ± 0.25	0	0.02 ± 0.15	0.07 ± 0.25	0	0
	*Brachionus plicatilis*	0.08 ± 0.27^a^	0.04 ± 0.19	0.10 ± 0.33	0	0.05 ± 0.21^b^	0	0.08 ± 0.27	0
	*Gammarinea* sp. (larva)	0.14 ± 0.67	0.09 ± 0.55	0.05 ± 0.58	0.08 ± 0.17	0.11 ± 0.47	0.03 ± 0.18	0	0.05 ± 0.32
	*Gammarinea* sp. (adult)	0.17 ± 1.03	0.09 ± 0.41	0.01 (n = 1)	0.08 ± 0.89	0.04 ± 0.25	0.01 ± 0.11	0	0.03 ± 0.23
	Other	0.18 ± 0.57	0.55 ± 1.81	00.11 ± 0.35	0.06 ± 0.24	0.08 ± 0.29	0.07 ± 0.25	0	0.03 ± 0.23
	Total	4.31 ± 4.42	4.18 ± 4.91	2.8 ± 4.75	7.2 ± 6.59	0.73 ± 2.97	1.16 ± 3.29	1.36 ± 0.23	1.58 ± 2.78
Intensity	Parasites	Summer	Autumn	Winter	Spring	Summer	Autumn	Winter	Spring
	*Mesanophrys* sp.	0.01 ± 0.09	0.02 ± 0.15	0	0.30 ± 0.44	0.01 ± 0.09	0.02 ± 0.15	0	0.30 ± 0.47
	*Pleuronema* sp.	0.02 ± 0.12^a^	0.38 ± 0.49^a^	1.25 ± 0.97^a^	0.84 ± 0.99^a^	0.02 ± 0.12^b^	0.38 ± 0.48^b^	1.25 ± 0.9^b^	0.84 ± 0.99^b^
	*Trichodina* sp.	2.25 ± 0.62	1.00(n = 1)	3.00 n = 1))	1.25 ± 0.46	0	0	0	0
	*Zoothamnium* sp.	4.00 ± 3.74^a^	3.33 ± 1.62^a^	2.98 ± 1.6	6.23 ± .3.98^a^	2.29 ± 1.38^b^	2.16 ± 1.3^b^	2.00 (n = 1)	2.27 ± 0.79^b^
	*Vortecella* sp.	0.04 ± 0.19	0.06 ± 0.38	0	0	0.04 ± 0.19	0.06 ± 0.38	0	0
	*Lecane* sp.	0.02 ± 0.15	0.07 ± 0.25	0	0	0.02 ± 0.15	0.07 ± 0.25	0	0
	*Brachionus plicatilis*	0.05 ± 0.21	0	0.08 ± 0.27	0	0.05 ± 0.21	0	0.08 ± 0.27	0
	*Gammarinea* sp. (larva)	1.36 ± 0.20	1.40 ± 0.25	1.17 ± 0.17	1.33 ± 0.33	1.56 ± 1.01	1.00 (n = 1)	0	2.01 (n = 1)
	*Gammarinea* sp. (adult)	1.80 ± 0.33	1.17 ± 0.17	1.40 ± 0.40	1.00 (n = 1)	1.00 (n = 1)	0	0	2.00 (n = 1)
	Other	1.50 ± 0.86	2.93 ± 3.19	1.00 ± 0.38	1.14 (n = 1)	1.69 ± 1.18	1.00 (n = 1)	0	2.00 (n = 1)
	Total	11.05 ± 6.31	10.36 ± 6.49	10.88 ± 3.89	12.09 ± 6.21	10 ± 6.41	8.82 ± 2.57	8.01 ± 5.1	13.41 ± 3.53

Data are presented as mean ± SD. Values with different superscripts in each season at the different sites indicate significant difference at p < 0.05. N; number of examined crabs, n; number of infected crabs.

The impacts of host length and weight on parasite dominance are illustrated in Table 7. At Site1, larger and taller crabs (Class II > 110.3 g and 56.02 cm) exhibited higher infection rates compared to smaller ones. In contrast, the biological factors in the sea showed different patterns, as taller crabs (Class II > 49.6 cm in length) and smaller crabs (Class I < 76.7 g in weight) recorded the highest prevalence (Table 7). The last biological factor represented by the host sex showed that males were more susceptible to the infection than females in both sites. There were no significant differences between the two sexes recorded in either abundance or intensity (Table 8).

**Table 7:** Dominance of different parasites in the blue crab *C. sapidus* in relation to its length and weight.

	Site1					Site2			
	Parasites	Length		Weight		Length		Weight	
		Class I N = 177	Class II N = 161	Class I N = 214	Class II N = 125	Class I N = 184	Class II N = 187	Class I N = 282	Class II N = 89
Prevalence (%)	*Mesanophrys* sp.	4.5	5.5	4.8	5.1	21.73	22.45	1	1.2
	*Pleuronema* sp.	4.2	5.8	5.01	7.6	0.2	0.4	0.2	0.5
	*Zoothamnium* sp.	5.62	5.24	5.54	6.2	5.97	18.18	7.81	25.8
	Vortecella sp.	4.0	4.0	2.0	6.6	1.0	0.5	0.1	0.6
	*Lecane* sp.	1.0	2.1	3.0	1	0.1	1.2	0.4	1
	*Brachionus plicatilis*	4.9	2.1	5.2	2.3	0.2	1.2	1.5	1.4
	*Gammarinea* sp. (larva)	9.01	10.32	7.94	9.1	0.54	6.95	28.2	3.56
	*Gammarinea* sp. (adult)	8.47	8.71	3.01	5.6	0.54	1.06	5.64	0.89
	Other	2.90	3.77	4.87	5.0	1.63	9.09	3.90	8.01
	Total	48.72	51.03	44.2	55.7	32.45	67.25	51.94	47.25
Abundance	Parasites	Class I	Class II	Class I	Class II	Class I	Class II	Class I	Class II
	*Mesanophrys sp.*	0.11 ± 0.34	0.15 ± 0.37	0.12 ± 0.34^a^	0.15 ± 0.38	0.06 ± 0.24	0.03 ± 0.18	0.04 ± 0.20^b^	0.06 ± 0.24
	*Pleuronema sp.*	0.74 ± 0.95^a^	0.99 ± 0.79	0.70 ± 0.93^a^	1.13 ± 0.72	0.61 ± 0.92^b^	0.39 ± 0.65	0.48 ± 0.85^b^	0.51 ± 0.63
	*Vortecella sp.*	0.02 ± 0.15	0.04 ± 0.29	0.05 ± 0.25	0.02 ± 0.15	0.01 ± 0.08	0.05 ± 0.30	0.02 ± 0.14	0.06 ± 0.39
	*Zoothamnium sp*	2.29 ± 3.32	2.80 ± 3.40	2.19 ± 3.19^a^	3.11 ± 3.56	0.02 ± 0.16	0.08 ± 0.29	0.18 ± 0.71^b^	0.54 ± 1.09
	*Lecane sp.*	0.06 ± 0.28	0.02 ± 0.14	0.07 ± 0.26	0.02 ± 0.19	0.03 ± 0.18	0.02 ± 0.15	0.02 ± 0.15	0.04 ± 0.19
	*Brachionus plicatilis*	0.08 ± 0.28	0.02 ± 0.19	0.15 ± 0.75	0.10 ± 0.33	0.03 ± 0.18	0.04 ± 0.19	0.05 ± 0.21	0
	*Gammarinea sp. (larva)*	0.10 ± 0.41	0.09 ± 0.32	0.10 ± 0.03	0.08 ± 0.03	0.01 ± 0.15	0.10 ± 0.43	0.06 ± 0.36	0.04 ± 0.21
	*Gammarinea sp. (adult)*	0.12 ± 0.53	0.07 ± 0.29	0.12 ± 0.51	0.06 ± 0.23	0.01 ± 0.08	0.04 ± 0.24	0.02 ± 0.19	0.02 ± 0.15
	Other	0.10 ± 0.33	0.35 ± 1.33	0.12 ± 0.45	0.38 ± 1.44	0.89 ± 1.64	1.2 ± 3.07	0.04 ± 0.22	0.09 ± 0.29
	Total	3.72 ± 7.06^a^	4.71 ± 7.86	3.64 ± 5.93	5.09 ± 7.37	0.01 ± 0.15^b^	0.10 ± 0.43	0.95 ± 3.45	1.46 ± 3.45
Intensity	Parasites	Class I	Class II	Class I	Class II	Class I	Class II	Class I	Class II
	*Mesanophrys sp.*	0.06 ± 0.24	0.03 ± 0.18	0.04 ± 0.20	1.08 ± 0.23	0.06 ± 0.24	0.03 ± 0.18	0.04 ± 0.20	0.06 ± 0.23
	*Pleuronema sp.*	0.61 ± 0.92^a^	0.39 ± 0.65	0.48 ± 0.85^a^	0.06 ± 0.23	0.61 ± 0.92^b^	0.39 ± 0.65	0.48 ± 0.85^b^	0.51 ± 0.63
	*Trichodina sp.*	1.89 ± 1.36	1.61 ± 1.65	1.11 ± 0.33^a^	0	1.00 (n = 1)	1.38 ± 0.87	1.11 ± 0.33^b^	1.80 ± 1.30
	*Vortecellasp.*	0.01 ± 0.08	0.05 ± 0.30	0.02 ± 0.14^a^	0.51 ± 0.6	0.01 ± 0.08	0.05 ± 0.30	0.02 ± 0.14^b^	0.06 ± 0.39
	*Zoothamnium sp.*	3.97 ± 3.53	4.75 ± 3.14	2.36 ± 1.17^a^	4.97 ± 3.27	2.27 ± 0.79	2.21 ± 1.30	2.36 ± 1.18^b^	2.09 ± 1.20
	*Lecane sp.*	0.03 ± 0.18	0.02 ± 0.15	0.02 ± 0.15	0.06 ± 0.39	0.03 ± 0.18	0.02 ± 0.15	0.02 ± 0.15	0.04 ± 0.19
	*Brachionus plicatilis*	0.03 ± 0.18^a^	0.04 ± 0.19	0.05 ± 0.21	0.04 ± 0.19	0.03 ± 0.18	0.04 ± 0.19	0.05 ± 0.21	0
	*Gammarinea sp. (larva)*	1.46 ± 0.66	1.17 ± 0.39	1.70 ± 0.94^a^	1.25 ± 0.46	2.00 (n = 1)	1.46 ± 0.88	1.70 ± 0.95^b^	2.00(n = 2)
	*Gammarinea sp. (adult)*	1.91 ± 1.04^a^	1.09 ± 0.30	1.50 ± 0.71	1.00 (n = 1)	1.00^b^ (n = 1)	1.50 ± 0.71	1.50 ± 0.71	1.00 (n = 1)
	Other	1.14 ± 0.36	2.30 ± 2.524	1.91 ± 1.22^a^	2.36 ± 2.75	1.33 ± 0.58	1.53 ± 1.07	1.91 ± 1.22^b^	1.00 (n = 1)
	Total	11.04 ± 8.56	13.06 ± 9.4	9.19 ± 5.94	11.33 ± 8.22	8.34 ± 2.96	8.61 ± 5.23	9.19 ± 5.94	7.56 ± 3.95

Data are presented as mean ± SD. Values with different superscripts in each season at the different sites indicate significant difference at p < 0.05 N; number of examined crabs, n; number of infected crabs.

**Table 8:** Dominance of different parasites in the blue crab *C. sapidus* in relation to its sex.

	Site1			Site2	
	Parasites	Male N = 190	Female N = 149	Male N = 226	Female N = 145
Prevalence (%)	*Mesanophrys sp.*	5.5	4.5	1.2	1.2
	*Pleuronema sp.*	5.6	4.4	2.1	2.3
	*Trichodina sp.*	7.36	3.90	4.42	4.75
	*Vortecella sp.*	6.2	3.8	2.6	1.6
	*Zoothamnium sp.*	5.78	1.08	14.15	13.96
	*Lecane sp.*	9.0	1	0.1	0.1
	*Brachionus plicatilis*	6.2	3.6	2.0	1.5
	*Gammarinea sp. (larva)*	6.31	8.72	20.34	15.2
	*Gammarinea sp. (adult)*	6.84	6.04	0.88	1.12
	Other	4.15	2.7	5.75	4.82
	Total	62.04	37.8	53.54	46.45
Abundance	Parasites	Male	Female	Male	Female
	*Mesanophrys sp.*	0.13 ± 0.35	0.13 ± 0.36	0.06 ± 0.23	0.03 ± 0.17
	*Pleuronema sp.*	0.86 ± 0.87	0.85 ± 0.89	0.51 ± 0.81	0.46 ± 0.79
	*Trichodina sp.*	0.15 ± 0.73	0.12 ± 0.46	0.05 ± 0.26	0.05 ± 0.36
	*Vortecella sp.*	0.05 ± 0.24	0.03 ± 0.20	0.04 ± 0.28	0.02 ± 0.12
	*Zoothamnium sp.*	2.46 ± 3.12	2.62 ± 3.64	0.31 ± 0.87	0.20 ± 0.78
	*Lecane sp.*	0.05 ± 0.29	0.01 ± 0.08	0.01 ± 0.12	0.05 ± 0.21
	*Brachionus plicatilis*	0.06 ± 0.27	0.05 ± 0.21	0.04 ± 0.20	0.02 ± 0.15
	*Gammarinea sp. (larva)*	0.08 ± 0.34	0.11 ± 0.41	0.05 ± 0.25	0.07 ± 0.42
	*Gammarinea sp. (adult)*	0.08 ± 0.35	0.11 ± 0.51	0.02 ± 0.18	0.02 ± 0.18
	Other	0.23 ± 0.90	0.21 ± 1.02	0.04 ± 0.23	0.06 ± 0.26
	Total	4.15 ± 7.46	4.51 ± 7.80	1.13 ± 3.43	0.95 ± 3.43
Intensity	Parasites	Male	Female	Male	Female
	*Mesanophrys sp.*	0.06 ± 0.23	0.03 ± 0.17	0.06 ± 0.23	0.03 ± 0.17
	*Pleuronema sp.*	0.51 ± 0.81	0.46 ± 0.78	0.51 ± 0.80	0.46 ± 0.79
	*Trichodina sp.*	2.00 ± 1.96	1.38 ± 0.87	1.20 ± 0.42	1.75 ± 1.50
	*Vortecella sp.*	0.04 ± 0.28	0.02 ± 0.12	0.04 ± 0.28	0.02 ± 0.12
	*Zoothamnium sp.*	4.48 ± 2.92	4.19 ± 3.79	2.22 ± 1.04	2.23 ± 1.54
	*Lecane sp.*	0.01 ± 0.12	0.05 ± 0.21	0.01 ± 0.12	0.05 ± 0.21
	*Brachionus plicatilis*	0.04 ± 0.20	0.02 ± 0.15	0.04 ± 0.20	0.02 ± 0.15
	*Gammarinea sp. (larva)*	1.33 ± 0.49	1.31 ± 0.63	1.22 ± 0.44	2.00 ± 1.22
	*Gammarinea sp. (adult)*	1.23 ± 0.59	1.89 ± 1.05	1.00 (n = 1)	2.00 (n = 1)
	Other	1.88 ± 1.83	1.94 ± 2.56	1.62 ± 1.19	1.29 ± 0.49
	Total	11.58 ± 9.49	11.04 ± 10.36	7.92 ± 4.73	9.85 ± 6.22

Data are presented as mean ± SD.

**Figure 8:**
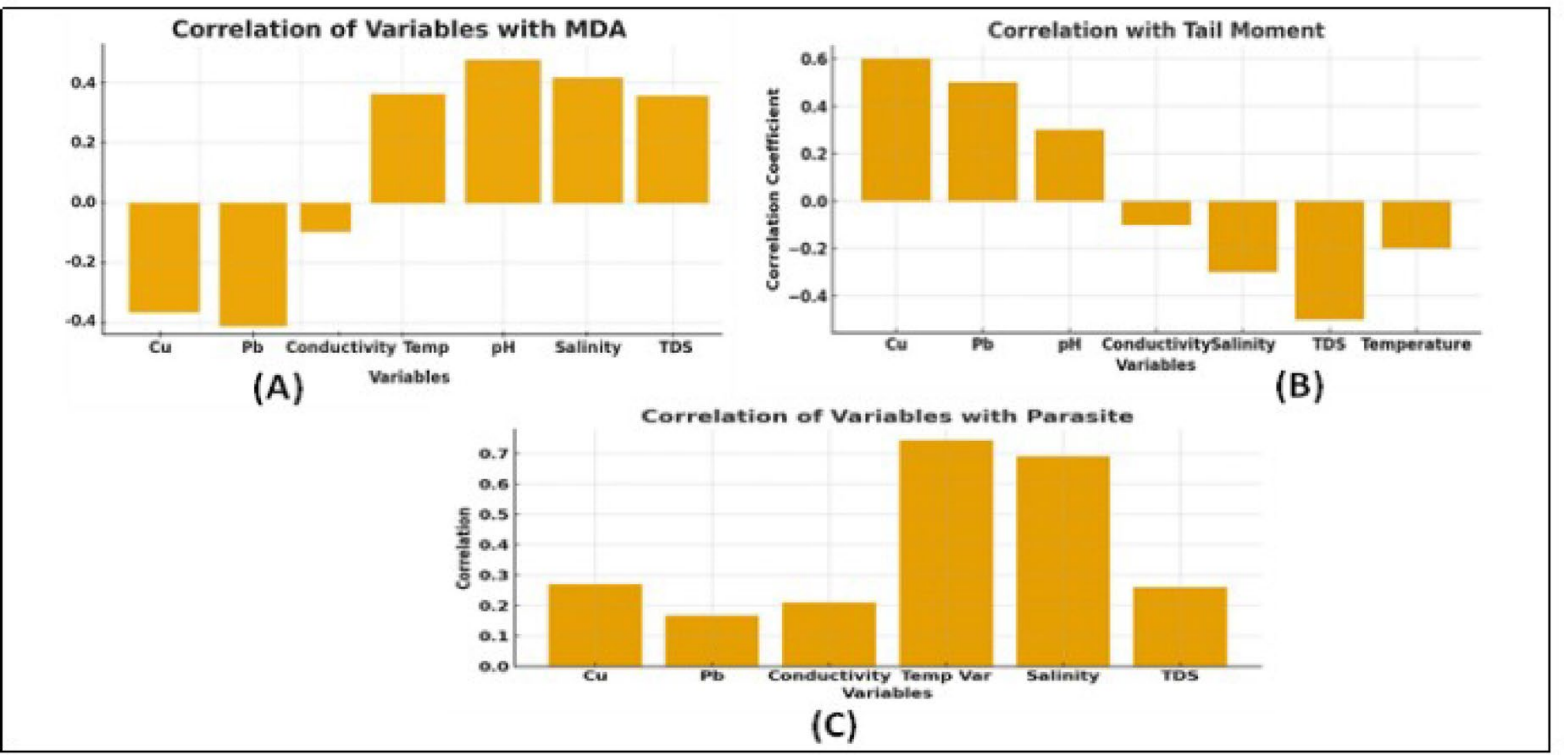
The impact of heavy metals and physicochemical parameters on** (A)** MDA. **(B)** Tail moment of comet assay. **(C)** Parasites.

The impact of physiochemical parameters and heavy metal on MDA, tail moment and parasites are represented in (Fig. 8). The result demonstrated a negative correlation between heavy metals and MDA levels, whereas a positive correlation between most physicochemical parameters with MDA levels except for conductivity (Fig. 8A). In addition, there were a positive correlation between heavy metals and tail moment, while a negative correlation between physiochemical parameters and tail moment except for pH (Fig. 8B). Regarding the number of parasites, both heavy metals and physicochemical parameters revealed positive correlations with parasites (Fig. 8C).

## Discussion

Environmental pollutants can greatly affect aquatic health. Due to human activities, aquatic ecosystems have become polluted with large quantities of inorganic anions and heavy metals. The main sources of this contamination are industrial processes, petroleum spills, and wastewater discharge of human and livestock. Hence the two studied sites have been chosen based on being different in the prior parameters.

The observed differences between El Bogus and El Matar can be largely attributed to their distinct ecological settings. El Bogus is more directly influenced by its hydrological connection to the lake, which facilitates continuous water exchange and may enhance the transport and accumulation of contaminants originating from surrounding areas. In contrast, El Matar is characterized by relatively different hydrodynamic conditions and varying degrees of exposure to anthropogenic activities, including human settlement and potential industrial inputs. These environmental gradients likely contribute to the spatial variation in pollution load and, consequently, the biological responses observed in the studied organisms. Therefore, site-specific ecological characteristics should be considered a key driver shaping the differences recorded between the two locations^36^

Physicochemical parameters showed an acidic pH at site 1 and an alkaline pH at site 2. This may be attributed to its direct connection with Lake Manzala. According to El-Serafy et al.^37^, the pH values of Lake Manzala are generally alkaline due to high photosynthetic activity and drainage rich in bicarbonates. Temperature was a key differentiating factor. It exhibited seasonal change ranging from 18.6 to 28.7 °C. Temperature affects water chemistry, biological activity, and related parameters such as pH, conductivity, and dissolved oxygen^38^. Salinity was observed to be higher during winter than in summer, which may be explained by the reduced freshwater inflow and lower evaporation rates during the colder months. A similar seasonal pattern was described along the Egyptian Mediterranean coast, where salinity variations are mainly controlled by temperature and freshwater discharge^39^.

Conductivity and total dissolved solids were consistently lower at site 1 than at site 2 due to the inflow of low-salinity drainage water^40^. These findings align with Elgendy et al.^41^, who reported seasonal 26.63–37.00 PSU salinity, and with Abd El-Fattah et al.^42^, who noted that conductivity and TDS increase with salinity. Total Dissolved Solids (TDS) represent the overall concentration of dissolved inorganic salts and minor organic matter in water, including key cations and anions. Measured in mg/L or ppm, TDS is a critical indicator of water quality, affecting taste, conductivity, and suitability for drinking or irrigation^43^.

Heavy metals, including Cu, Pb, and Cd, are among the most hazardous and prevalent inorganic environmental pollutants, primarily originating from industrial discharges and mining activities. Crabs can accumulate heavy metals in their tissues through absorption via the gills, a process influenced by metal concentrations in water and sediment, as well as the specific type of metal^44^.

Fortunately, Cd was not detected in water, sediment, or crab muscle tissues at both sites during the study period. Several studies reported variable findings regarding Cd levels, along the Mediterranean coast and in Lake Manzala, with some detecting its presence and others not^45–47^. Such discrepancies may be attributed to the difference in sampling locations or environmental conditions that affect heavy metal accumulation^48,49^. Moreover, it explained the government’s efforts in cleaning Lake which recently carried out.

Cu and Pb concentrations varied significantly with seasons in the current study. The highest concentration was recorded in spring. This was disagreed with EL-Fahla et al.^50^, who registered the highest level of Cu concentration in autumn. This may be attributed to the difference in longitude, latitude, and physicochemical parameters of the aquatic environment. The present study registered higher levels of Cu and Pb at site 2 than at Site1. This mainly resulted from the continuous land-based discharges (industrial, agricultural, and municipal), in addition to re-mobilization from sediments and shipping-related inputs, as previously reported by El-Serehy et al.^51^

Bioaccumulation Factor (BAF) is a ratio that describes the accumulation of a chemical substance (such as heavy metals) in an organism relative to its concentration in the surrounding environment, typically water (BCF) and sediment (BSAF). When BAF is less than 1, it means that there is not enough or too little accumulation, or the sedimentation index exceeds one, it indicates bioaccumulation^52^. In the present study, BASF of Cu was less than one. Alternatively crabs exhibited bioaccumulation of Cu from water, as its BCF exceeded one. This suggests that Cu present in the aqueous phase are more biologically available and are taken up more efficiently by the crabs than those bound to sediments. Also, dissolved metals in water are more readily absorbed via physiological interfaces, leading to elevated BCF values. Additionally, sediments serve as a major reservoir for heavy metals through adsorption onto organic matter and fine particles, this strong binding limits metal mobility. So, metal bioaccumulation is controlled not only by environmental concentrations but also by differences in bioavailability and exposure pathways^53^. Generally, the values of BCF, and BASF were higher in Cu than Pb, this may be Cu is an essential metal homeostatically regulated by the organism, Cu uptake in crabs may occur via both environmental exposure and dietary ingestion with food acting as a significant source of copper accumulation^54^ To assess the potential risks of heavy metals in the muscles of *C. sapidus*, the estimated metal concentrations were compared with international standards set by the Food and Agriculture Organization (FAO) and the World Health Organization (WHO) (Table 9). Our results indicate that Cu and Cd levels were within the permissible limits and posed no risk to human health. Although Pb concentrations remained within the permissible limits established by FAO, the recorded values exceeded WHO guideline levels, which may indicate a potential public health concern, particularly under conditions of frequent seafood consumption. Chronic exposure to lead, even at relatively low concentrations, may result in neurological, renal, and developmental disorders. Therefore, continuous monitoring of heavy metal accumulation in edible crab tissues is recommended to minimize potential health risks to consumers, especially vulnerable groups such as children and pregnant women^55^

**Table 9:** A comparison between the permissible limits (ppm) for heavy metals in food and in *C. sapidus*.

Metal	Cu	Pb	Cd
WHO (1989)	30	2.0	1
FAO (1983)	10–100	0.5–6	0.05–5.5
Present study			
Site1	1.97 ± 0.03	2.77 ± 0.01	0
Site2	3.11 ± 0.05	3.37 ± 0.03	0

Environmental pollutants can induce the generation of reactive oxygen species (ROS). When an imbalance occurs between the accumulations of ROS in cells and tissues and the generation of antioxidants that detoxify these reactive products, it is called oxidative stress^56–58^. This stress led to the damage of cellular macromolecules such as DNA, proteins, and lipids^59,60^.

MDA levels and the comet assay can be used as oxidative stress biomarkers. MDA is a byproduct formed during the lipid peroxidation of polyunsaturated fatty acids^61^. In the present study, MDA recorded its highest levels in the summer at both sites. This increase is likely attributable to elevated intensified environmental pollution as well as physicochemical conditions of the sea during the summer season. Pereira et al.^62^ concluded that deviations from the optimal temperature, whether higher or lower, increase oxidative stress and MDA production by disrupting cellular metabolism and enhancing ROS generation.The elevated MDA levels observed during summer may not be attributed solely to pollution stress. Increased water temperature during summer can enhance metabolic activity and reactive oxygen species (ROS) production, thereby contributing to oxidative stress and lipid peroxidation.^63,64^

Other physicochemical parameters, such as salinity and TDS, exhibited positive correlation with MDA in the present study. It can be hypothesized that an elevation in salinity and TDS imposes osmotic and ionic stress on the crab, leading to increased MDA production because of oxidative damage.

Olakolu et al.^65^ observed elevated MDA levels in crab samples from the polluted sites, attributing this to contamination in the lagoon environment. This was consistent with Janssens et al.^66^ who reported that certain invertebrates have developed strong tolerance mechanisms to heavy metals, often through the overexpression of protective proteins such as metallothionein. These proteins bind and sequester heavy metals, thereby reducing their bioavailability and mitigating oxidative damage. As a result, MDA production remains low even under elevated Cu or Pb exposure. Furthermore, evolutionary adaptation in some populations enables the maintenance of low oxidative stress markers, such as MDA, despite inhabiting metal-rich environments^67^.

Comet assay can assess levels of oxidative stress by measuring DNA damage^68^. The incidence of DNA damage in marine organisms is of particular concern, as DNA strand breaks can result from interactions with free radicals, organic and inorganic pollutants, heavy metals, and the formation of DNA adducts^68,69^. Numerous studies have confirmed that DNA strand breakage is a sensitive and reliable endpoint for assessing genotoxicity^68,70^. If DNA damage is not properly repaired, it can adversely affect DNA function^71,72^.

In the present study, comet assay results indicated DNA damage at site 1 compared to site 2 during the study period. This was in parallel with higher MDA levels at this site. These results align with Pisoni et al.^69^ and Al-Mashhadani et al*.*^73^. They reported DNA single-strand breaks in all tissues of *C. japonica* exposed to the heavy metals Cu, Pb, and Cd, as well as other environmental pollutants. In their study, DNA strand breakage was positively correlated with metal concentrations, thereby supporting the observations of the present study.

In the present work, there was a positive correlation between pH and tail moment in the comet assay. Higher pH values increase DNA unwinding, making strand breaks more detectable and resulting in higher tail moments^74^. A positive correlation between tail moment and heavy metals like Cu and Pb has been previously reported. As the increased exposure to these metals leads to the production of reactive oxygen species (ROS), resulting in more DNA strand breaks and, consequently, higher tail moments. This indicates greater genotoxicity from copper and lead^75^.

The parasites isolated from* C. sapidus* in the present study included protozoans, particularly ciliates, such as *Mesanophrys* sp. This finding is consistent with Xiao et al*.*^76^, who reported similar protozoan parasites in the swimming crabs, *Portunus trabeculate*. Additionally, species of the genus *Pleuronema*, which have been identified in a variety of aquatic environments, were also recorded in the current study. Pan et al.^77^ and Liu et al.^78^ described it among new marine scuticociliates. *Trichodina* sp. and *Zoothamnium* sp. were also reported in the present study. These genera were also reported in the blue and mud crabs^79^.

Rotifers such as *Lecane* sp. and *B. plicatilis* were also detected in the present study*.* These species previouslyassociated with the successful larval rearing and shellfish, including shrimp and crabs^80^. Additionally, nematodes such as *Gammarinema* sp. were also detected. Westerman et al.^81^ stated that this species inhabit the gill chambers of some decapods such as crabs. These findings highlight the diversity of parasitic and commensal organisms associated with *C. sapidus* and underscore the ecological complexity of host-parasite interactions.

In the present study, the overall prevalence at site 1 was about 62.8%, with an abundance of 8.57 ± 4.18 and an intensity of 22.18 ± 8.64. while at site 2 it was 38.4%, with an abundance of 4.15 ± 19.75 and an intensity of 4.35 ± 0.736. Ciliophora was the most frequent, collectively accounting for roughly 60% of infections. *Zoothamnium* sp. was the most prevalent parasite recorded in *C. sapidus.* Similar result previously reported by Muttaqin et al.^82^, as they considered *Zoothamnium* sp. was common on various crustaceans, including the crab *Scylla serrata.*

Larger crabs (in the term of weight and length) showed higher infection rates (55.7% and 51.03%, respectively) at Site1 than smaller ones. This agrees with Childers et al.^83^, who reported a 41% prevalence of *Nadelspora canceri* on the larger Dungeness crab. This was not the case at Site2, as the larger crabs in length and small in weight showed higher infection rates (67.25% and 51.94%, respectively). The common factor between the two sites is the length, this may attributed to their longer exposure time and wider surface area for attachment. The higher prevalence of parasitic infection observed in male crabs may be related to behavioral and physiological differences between sexes. Male crabs generally exhibit greater mobility, wider foraging activity, and increased territorial or mating behaviors, which may increase their exposure to infective stages of parasites. In addition, sex-related differences in immune responses and energy allocation could contribute to higher susceptibility in males^84^.It may be attributed to the immune condition of the crabs and the environmental factors that differed from Site1. This agreed with Shields^85^ who found that the prevalence of *Hematodinium* infection in crabs was influenced by carapace size, with higher infection in medium sized individuals that due to variations in immune condition and exposure to environmental factors.

A significant sex-based difference in the parasite’s infection was reported by Ugbonmeh et al. (2015). This finding disagreed with the present study, as the observed difference in parasite dominance between crab sexes was not statistically significant (p = 0.1). A similar result recorded by Ruhay and Ibrahim^86^ in the blue crab *C. sapidus* in East Mediterranean Sea. Anderson^87^, previously stated that variations in parasite infection are likely influenced by ecological factors such as host parasite interactions and abiotic conditions rather than host sex. Therefore, the present study recorded a positive correlation between parasites and physicochemical parameters such as temperature, salinity, conductivity, and TDS. According to Mehana et al.^88^, higher values of these parameters can increase the prevalence, development, and transmission of parasites infecting invertebrates. The warmer temperature accelerates parasite life cycles, while higher salinity and TDS favor certain parasite stages or species in aquatic invertebrates. A positive correlation between parasites and heavy metals Cu and Pb) were also recorded in the present study this agreed with Sures et al.^89^, who documented that the parasites can accumulate heavy metals in their tissues, acting as metal sinks which protect the host by reducing the bio available metal load.The relationship between parasitic infection and environmental pollution is complex and remains a matter of debate. Previous studies have reported both positive and negative associations depending on parasite species, host characteristics, pollutant type, and environmental conditions^90^.

## Conclusion

The present study demonstrates that *C. sapidus* can effectively reflect the combined effect of environmental pollutants on the aquatic organisms’ health. Moreover, MDA levels, DNA damage, and parasitic infections were more closely associated with seasonal variations in physical and chemical parameters than heavy metals. These findings underscore the importance of integrated bio-monitoring approaches that consider both chemical and biological stressors to gain a comprehensive understanding of ecosystem health.

## Data Availability

The datasets used and/or analysed during the current study available from the corresponding author on reasonable request.

## References

[1] Aurelle, D. et al. Biodiversity, climate change, and adaptation in the Mediterranean. *Ecosphere* **13**(4), e3915. 10.1002/ecs2.3915 (2022).

[2] El-Ghobashy, A. E., Abdallah, M. M. & El-Hawary, W. F. Assessment of fishery resources and catch composition along the Egyptian Mediterranean coast. *Egypt. J. Aquatic Biol. Fish.* **25**(3), 123–138 (2021).

[3] Zubair, M. et al. Sodium arsenite toxicity on hematology indices and reproductive parameters in Teddy goat bucks and their amelioration with vitamin C. *Environ. Sci. Pollut. Res.* **27**, 15223–15232 (2020). 10.1007/s11356-020-08049-z 32072415

[4] Wahba, S. M. A consumption-based approach to trace the effects of income inequality on water pollution responsibility in Egypt : An internal grey water footprint perspective. *Ecol. Econ.* **227**, 108404. 10.1016/j.ecolecon.108404 (2025).

[5] Luo, P. et al. Historical assessment and future sustainabilitychallenges of Egyptian water resources management. *J. J. Clean*10.1016/j.jclepro.2020.121154 (2020).

[6] Radhakrishnan, K., Prakasheswar, P., Pradhap, D., Akramkhan, N., Rajkumar, A., Maheswaran, S, P., Krishnakumar, S. A baseline study on polycyclic aromatic hydrocarbons (PAHS) in surface sediments,The Journal of Kerala Estuaries, West Coast of India (2023). 10.1016/j.totert.2023.100055

[7] Xu, W. et al. Effects of GSH1 and GSH2 gene mutation on glutathione synthetases activity of *Saccharomyces cerevisiae*. *Protein J.* **36**(4), 270–277. 10.1007/s10930-017-9731-0 (2017). 28669025 10.1007/s10930-017-9731-0

[8] Boldrocchi, G. et al. Legacy and emerging contaminants in the endangered filter feeder basking shark Cetorhinus maximus. *J. Mar. Pollut. Bull.* **176**, 113466. 10.1016/j.marpolbul.2022.113466 (2022). 10.1016/j.marpolbul.2022.113466 35219080

[9] Zicarelli, G., Multisanti, C. R., Falco, F. & Faggio, C. Evaluation of Toxicity of Personal Care Products (PCPs) in Freshwaters:Zebrafish as a Model. *Environ. Toxicol. Pharmacol* **94**, 10392. 10.1016/j.etap.2022.103923 (2022). 10.1016/j.etap.2022.103923 35772612

[10] Gelany, A. Enhancing the preservation of submerged marble artifacts with cod liver oil in Alexandria, Egypt. *Aswan Univ. J. Environ. Stud. AUJES* **6**(1), 51–62. 10.21608/aujes.2025.345451.1313 (2025).

[11] Wahaab, R. A. & Badawy, M. I. Water quality assessment of the River Nile system:An overview. *Biomedicaland Environ. Sci.* **17**, 87–100 (2004). 15202868

[12] Zahran, M. A., El-Amier, Y. A., Elnaggar, A. A., Abd El-Azim, H. & El-Alfy, M. A. Assessment and distributionof heavy metals pollutants in Manzala Lake, Egypt. *J. Geosci. Environ. Protect.* **3**, 107–122. 10.4236/gep.2015.36017 (2015).

[13] Álvarez-Ruiz, R., Picó, Y. & Campo, J. Bioaccumulation of emerging contaminants in mussel (*Mytilus galloprovincialis*): Influence of microplastics. *Sci. Total Environ.* **796**, 149006. 10.1016/j.scitotenv.2021.1490063 (2021). 34328891 10.1016/j.scitotenv.2021.149006

[14] Impellitteri, F. et al. Exploring the impact of contaminants of emerging concern on fish and invertebrates physiology in the Mediterranean Sea. *Biology (Basel)* **12**, 767. 10.3390/biology12060767 (2023). 37372052 10.3390/biology12060767 PMC10295567

[15] Sandys, O. & TeVelde, A. Raising the alarm: Environmental factors in the onset and maintenance of chronic (low-grade) inflammation in the gastrointestinal tract. *Dig. Dis. Sci.*10.1007/s10620-021-07327-1 (2022). 34981314 10.1007/s10620-021-07327-1

[16] Xu, X., Nie, S., Ding, H. & Hou, F. F. Environmental pollution and kidney diseases. *Nat. Rev. Nephrol.* **14**(5), 313–324. 10.1038/nrneph.2018.11 (2018). 29479079 10.1038/nrneph.2018.11

[17] Zheng, S. et al. Effects of environmental contaminants in water resources on nonalcoholic fatty liver disease. *Environ. Int.* **154**, 106555. 10.1016/j.envint.2021.106555 (2021). 33857709 10.1016/j.envint.2021.106555

[18] Zhang, H. C., Shl, C. Y. & Sun, L. Q. Toxic effects of ionic liquid 1-octyl-methylimidazolium bromide on the antioxidant defense system of freshwater planarian journal Dugesia japonica. *Toxicol. Ind. Health.* **32**, 1675–1683 (2016). 25812565 10.1177/0748233715573692

[19] Zhao, Y., Wang, S., Wu, J. & Ma, W. Effects of heavy metals from soil and dust source on DNA damage in Leymus chinensis leaves. *PLoS ONE*10.1371/journal.pone.0166522 (2017). 27935969 10.1371/journal.pone.0166522 PMC5147816

[20] Morales, M. E. et al. Heavy metal exposure influences double strand break DNA repair outcomes. *J. Pone*10.1371/0151367 (2016). 10.1371/journal.pone.0151367 PMC4788447 26966913

[21] Slavik, J.A and Nas, D. F." Diseases caused by protozoa". In: Provenzano Journal (Ed.), the Biology of Crustacea 6. Pathobiology, Academic Press, New York (2017). 10.28 (2):200–205

[22] Hatcher, J. et al. Indirect effects of parasites in invasions. *J. Appl. Ecol.* **10**(58), 2999–3012 (2012).

[23] Griffiths, K. M., Carron-Arthur, B., Parsons, A. & Reid, R. Effectiveness of programs for reducing the stigma associated with mental disorders: A meta-analysis of randomized controlled trials. *World Psychiatry* **13**(2), 161–175. 10.1002/wps.20129 (2014). 24890069 10.1002/wps.20129 PMC4102289

[24] Kostić, J. & Ilić, T. DNA damage induced by parasitic infections in humans and animals. *Acta Vet.* **75**(1), 45–58. 10.1016/j.actavet.2025.01.005 (2025). 10.1016/j.cimid.2025.102337 40220655

[25] Kampouris, T. E., Porter, J. S. & Sanderson, W. G. Callinectes sapidus Rathbun, 1896 (Brachyura: Portunidae): Assessment of diet, foraging behaviour, and ecological-economic impacts in Thermaikos Gulf, NW Aegean Sea, Greece. *Crustacean Res.* **48**, 23–37. 10.18353/crustacea.48.0_23 (2019).

[26] Marchessaux, G., Bizzarri, S., Marsiglia, N., Ponzè, N. & Sarà, G. The use of an unmanned aerial vehicle to investigate habitat use and behavior of invasive blue crab in Mediterranean microhabitats. *Mediterr. Mar. Sci.* **24**(2), 229–240. 10.12681/mms.31332 (2023).

[27] Nehring, S., G. Speckels and J. Albersmeyer. The American blue crab Callinectes sapidus Rathbun on the German North Sea coast: Status quo and further p (2008). 10.1007/BF03043867

[28] Streftaris, N. & Zenetos, A. Alien marine species in the Mediterranean-the 100 ‘worst invasives’ and their impact. *Mediterr. Mar. Sci.* **7**, 87–118. 10.12681/mms.180 (2006).

[29] Eja, M. E., Ogri, O. R. A. & Arikpo, G. E. Bioconcentration of heavy metals in surface sediments from the great KWA rivers estuary, Calabar, south Eastern Nigeria. *J. Nigerian Environ. Soc.* **2**, 247–256 (2003).

[30] Gad El-Hak, H. N. G., Ghobashy, M. A., Mansour, F. A., El-Shenawy, N. S. & El-Din, M. I. S. Heavy metals and parasitological infection associated with oxidative stress and histopathological alteration in the Clarias gariepinus. *Ecotoxicology* **31**(7), 1096–1110. 10.1007/s10646-022-02569-9 (2022). 35840811 10.1007/s10646-022-02569-9 PMC9458584

[31] Ponnusamy, K., Sivaperumal, P., Suresh, M., Arularasan, S., Munilkumar, S., Pal, A. K. Heavy metal concentration from biologically important edible species of bivalves (Perna viridis and Modiolus metcalfei) from Vellar estuary, south east coast of India (2014). 10.4172/2155-9546.1000258

[32] Adolfsson-Erici, M., Åkerman, G. & Mclachlan, M. S. Measuring bioconcentration factors in fish using exposure to multiple chemicals and internal benchmarking to correct for growth dilution. *Environ. Toxicol. Chem.* **31**, 1853–1860. 10.1002/etc.1897 (2012). 22639194 10.1002/etc.1897

[33] Messick, G. A. *Hematodinium perezi* infections in adult and juvenile blue crabs *Callinectes sapidus* from coastal bays of Maryland and Virginia, USA. *Dis. Aquat. Org.* **19**, 77–82 (1994).

[34] Sachs, R., Cumberlidge, N. The dwarf river crab Liberonautes latidactylus nanoides Cumberlidge and Sachs 1989, from Liberia- a new second intermediate host of Paragonimus uterobilateralis. Ann Trop Med Parasitol. 42(1) 2052862

[35] Song, Li., Wenbao Zhuang, Xiaochen Feng, Alan Warren, Jun Gong, Microorganisms , 13(2), 240 (2025); 10.3390/microorganisms13020240 10.3390/microorganisms13020240 PMC11857559 40005607

[36] El-Alfy, M. A., Elfanagily, B. A., Zyadah, M. A. & El-Emam, D. A. CA-Markov chain for simulation and prediction of LULC and assessing the status of water pollution in Manzala Lake after recent development. *J. Coastal Conserv.* **28**, 4. 10.1007/s11852-023-01005-2 (2024).

[37] El-Serafy, M., Abd El-kader, A., A. and Zaky, M., M. Alfarama Journal of Basic Applied Sciences (2022), 10.21608/AJBAS.2022.115353.1084

[38] Ismail, A. & Hettiarachchi, H. Environmental damage caused by wastewater discharge into the Lake Manzala in Egypt. *Am. J. Biosci. Bioeng.* **5**(6), 141–150. 10.11648/j.bio.20170506.14 (2017).

[39] Mohamed, E. E., Dabbous, A. S., Maiyza, H. I. & El-Geziry, T. M. Long-term variations in the salinity off the Egyptian Mediterranean coast. *Blue Econ.* **1**(2), Article 6. 10.57241/2805-2994.1012 (2023).

[40] El-Gawady, A. M. Assessment of some water quality characteristics and determination of some heavy metals in Lake Manzala. *Egypt Egypt. J. Aquatic Biol. Fish.* **12**(2), 133–154. 10.2160/ejabf.2008.1998 (2008).

[41] Elgendy, Sarah, A., Fedekar, F., Mohamed Ismail1, Khaled M. Abdelsalam2 Alfarama., Journal of Basic & Applied Sciences (2025). 10.21608/AJB A AS.2024.331976.1236

[42] Abd El-Fattah, H., El-Sayed, M. & El-Gayar, A. Chemical characteristics of the surface water around Ras El-Bar. *J. Environ. Stud. Eng.*10.21608/joese.2020.147763 (2020).

[43] Abbas, M. et al. Delineation of water quality aspects through water quality index using GIS and statistical approach in Faisalabad. *J. Glob. Innov. Agric. Sci.* **11**(1), 83–89. 10.22194/JGIAS/11.1.01 (2023).

[44] Agbugui, M. O. & Abe, G. O. Heavy metals in fish: bioaccumulation and health. *Br. J. Earth Sci. Res.* **10**(1), 47–66. 10.37745/bjesr.2013 (2022).

[45] Abd El-Kader, A. I., Bahnasawy, M., Zaky, M. & El-Serafy, M. A. Assessment of heavy metal concentration in water and the Nile tilapia of Lake Manzala, EL-Kapoty, Egypt. *Egypt. J. Aquatic Biol. Fish.* **26**(5), 137–147. 10.2160/ejabf (2022).

[46] El-kader, A. Assessment of heavy metal concentration in water and the Nile tilapia of Lake Manzala, EL-Kapoty, Egypt. *Egypt. J. Aquatic Biol. Fish.* **26**(5), 137–147. 10.21608/ejabf.2022.258909 (2022).

[47] Olgunoğlu, M. P., Artar, E. & Olgunoğlu, I. A. Comparison of heavy metal levels in muscle and gills of four benthic fish species from the Northeaste rn Mediterranean Sea. *Pol. J. Environ. Stud.* **24**(4), 1743–1748. 10.1524/pjoes/38972 (2015).

[48] Islam, S., Idris, A. M. & Islam, A. R. MTl. Hydrological distribution of physicochemical parameters and heavy metals in surface water and their ecotoxicological implications in the Bay of Bengal coast of Bangladesh. *Environ. Sci. Pollut. Res.* **28**, 68585–68599. 10.1007/s11356-021-15353-9 (2021). 10.1007/s11356-021-15353-9 34275081

[49] Uchenna, S.O. Assessment of Water Quality of Ndakaini Dam in Murang’a County.Kenya. Doctoral dissertation, University of Nairobi )2022)

[50] El-Fahla, N. A., Saad El-Din, M. I. & Abd El Mageed, Y. S. M. Gene expression analysis, biochemical and histological alterations in the Nile Tilapia (Oreochromis niloticus) exposed to Bisphenol A: The protective role of proanthocyanidin. *Egypt. J. Aquatic Biol. Fish.* **28**(5), 429–455 (2021).

[51] El-Serehy, H. A., Aboul-Ezz, S. M., Khalil, M. T. & Abdallah, M. A. M. Heavy metals contamination of a Mediterranean coastal ecosystem, Eastern Nile Delta, Egypt. *Turk. J. Fish. Aquatic Sci.* **12**(4), 751–760. 10.4194/1303-2712-v12_4_03 (2012).

[52] Barhoumi, S., Messaoudi, I., Deli, T., Said, K. & Kerkeni, A. Cadmium bioaccumulation in three benthic fish species, Salaria basilisca, Zosterisessor ophiocephalus, and Solea vulgaris collected from the Gulf of Gabes in Tunisia. *J. Environ. Sci.* **21**, 980–984. 10.1016/S1001-0742(08)62371-2 (2009). 10.1016/s1001-0742(08)62371-2 19862966

[53] Rahman, M. M., Hossain, M. A., Jahan, S. A. & Begum, A. Assessment of heavy metals in pangasius and tilapia aquaculture and human consumption risk. *Aquac. Int.* **30**(3), 1407–1434. 10.1007/s10499-022-00903-w (2024).

[54] Linder, M. C. Copper homeostasis in mammals, with emphasis on secretion and excretion: A review. *Int. J. Mol. Sci.* **21**(14), 4932. 10.3390/ijms21144932 (2020). 32668621 10.3390/ijms21144932 PMC7403968

[55] Jaishankar, M., Tseten, T., Anbalagan, N., Mathew, B. B. & Beeregowda, K. N. Toxicity, mechanism and health effects of some heavy metals. *Interdiscip. Toxicol.* **7**(2), 60–72. 10.2478/intox-2014-0009 (2014). 26109881 10.2478/intox-2014-0009 PMC4427717

[56] Janion, K., Krysa, E., Górska, N., Malinowska, A. & Chabowski, A. Evaluation of malondialdehyde level, total oxidant/antioxidant status and oxidative stress index in colorectal cancer patients. *Metabolites* **12**(11), 1118. 10.3390/metabo12111118 (2022). 36422258 10.3390/metabo12111118 PMC9695970

[57] Kucharova, K., Barvíková, J., Höhne, S., Janurová, K. & Nešporová, O. M. Antioxidant defense system components and their relationship with anthropometric measures and lipid metabolism biomarkers in apparently healthy women. *Biomedicines* **11**(9), 2450. 10.1007/s00204-024-03903-2 (2019). 10.3390/biomedicines11092450 PMC10525661 37760891

[58] Pizzino, G. et al. Oxidative stress: Harms and benefits for human health. *Oxid. Med. Cell. Longev.* **2017**, 8416763. 10.1155/2017/8416763 (2017). 28819546 10.1155/2017/8416763 PMC5551541

[59] Jomova, K., Alomar, S. Y. & Nepovimova, E. Heavy metals: Toxicity and human health effects. *Arch. Toxicol.* **99**, 153–209. 10.1007/s00204-024-03903-2 (2025). 39567405 10.1007/s00204-024-03903-2 PMC11742009

[60] Lai, E., Horta, E., Dahlen, E. & Engel, C. Rules for somatic variants in AKT3, MTOR, PIK3CA, and PIK3R2. *Genet. Med.* **26**(11), 101214. 10.1016/j.gim.2024.101214 (2024). 39011768 10.1016/j.gim.2024.101214

[61] Cordiano, R. et al. Malondialdehyde as a potential oxidative stress marker for allergy-oriented diseases: An update. *Molecules*10.3390/molecules28165979 (2023). 37630231 10.3390/molecules28165979 PMC10457993

[62] Pereira, K. S. et al. Effect of Increased Salt Water Intake on the Production and Physicochemical Composition of Goat Milk in the Brazilian Semiarid. *Animals* **11**(9), 2667. 10.3390/ani11092642 (2021). 34573608 10.3390/ani11092642 PMC8468390

[63] Cominassi, L. et al. Metabolic rate increases with acclimation temperature and is associated with mitochondrial function in some tissues of threespine stickleback. *J. Exp. Biol.* **225**(21), jeb244659. 10.1242/jeb.244659 (2022). 36268761 10.1242/jeb.244659 PMC9687547

[64] Livingstone, D. R. Contaminant-stimulated reactive oxygen species production and oxidative damage in aquatic organisms. *Mar. Pollut. Bull.* **42**(8), 656–666. 10.1016/S0025 (2001). 11525283 10.1016/s0025-326x(01)00060-1

[65] Olakolu, F.C. Hassan A.A., K.O., Renner British JOURNAL OF SCIENCE JUNE,vol 5(2) lipid peroxidation and antioxidant biomarker activities as indicator of pollution (2012). 10.3153/JAEFR15009

[66] Janssens, T. K. S., Roelofs, D. & Van Straalen, N. M. Molecular mechanisms of heavy metal tolerance and evolution in invertebrates. *Insect Sci.* **16**, 3–18. 10.1111/j.1744-7917.2009.00249.x (2009).

[67] Coyle, P., Philcox, J. C., Carey, L. C. & Rofe, A. M. Metallothionein: The multipurpose protein. *Cell. Mol. Life Sci.* **59**(4), 627–647 (2002). 12022471 10.1007/s00018-002-8454-2 PMC11337511

[68] Rajasekhar, S. S. N., Chaturvedula, L., Adole, P. & Adole, P. S. Deoxyribose nucleic acid damage and its association with plasma malondialdehyde levels among patients with cervical cancer: A case-control study. *Cureus* **16**(1), e52600. 10.7759/cureus.52600 (2024). 38374844 10.7759/cureus.52600 PMC10875276

[69] Pisoni, M., Cogotzi, L., Frigeri, A., Corsi, I., Bonacci, S., Iacocca,A., Lancini, L., Mastrotoraro, F., Focardi, S., and Svelto, M., . DNA adducts, benzo(a)pyrene monooxy (2004). 10.1016/j.envres.2004.02.011 10.1016/j.envres.2004.02.011 15325877

[70] Olive, P. L. DNA damage and repair in inidividual cells: Applications of the comet assay in radiobiology. *Int. J. Radiat. Biol.* **75**, 395–405. 10.1080/095530099140311 (1999). 10331844 10.1080/095530099140311

[71] Almeida, J. A. et al. The use of the oxidative stress responses as biomarkers in Nile tilapia (*Oreochromis niloticus*) exposed to in vivo cadmium contamination. *Environ. Int.* **27**, 673–679. 10.1016/S0160-4120(01)00127-1 (2002). 11934117 10.1016/s0160-4120(01)00127-1

[72] Chapman, H. et al. Algebraic differentiation for fast sensitivity analysis of optimal flux modes in metabolic models. *Bioinformatics* **41**(6), 2185–2193. 10.1093/bioinformatics/btaf287 (2025). 10.1093/bioinformatics/btaf287 PMC12133274 40327453

[73] Al-Mashhadani, S. A. & Al-Mashhadani, H. M. Evaluation of malondialdehyde, C-reactive protein and DNA damage related with the smoking habit by comet assay in Iraq. *Int. J. Environ. Res. Public Health* **18**(12), 6353. 10.25258/ijddt.11.2.10 (2021).34208212

[74] Hermeto, L. C. et al. Evaluation of pH effects on genomic integrity in adipose-derived mesenchymal stem cells using the comet assay. *Genet. Mol. Res.* **14**(1), 339–348 (2015). 25729966 10.4238/2015.January.23.7

[75] Zhao, H., Huang, H. & Lin, S. Chemical approaches to angiogenesis in development and regeneration. *Methods Cell Biol.* **134**, 369–376. 10.1016/bs.mcb.2016.03.007 (2016). 27312498 10.1016/bs.mcb.2016.03.007

[76] Xiao, C. & Lai, D. Impact of oxidative stress induced by heavy metals on ovarian function. *J. Appl. Toxicol.* **45**(1), 107–116. 10.1002/jat.4664 (2024). 38938153 10.1002/jat.4664 PMC11634564

[77] Pan, X. et al. Morphology and phylogeny of four marine scuticociliates (Protista, Ciliophora), withdescriptions of two new species: Pleuronema elegans spec. nov. and Uronema orientalisspec nov.. *Acta Protozool.* **54**, 31–43. 10.4467/16890027AP.15.003.2190 (2015).

[78] Liu, M. et al. Integrative studies onthe taxonomy and molecular phylogeny of four new *Pleuronema* species (Protozoa,Ciliophora, Scuticociliatia). *Mar. Life Sci. Technol.* **4**, 179–200. 10.1007/s42995-022-00130-5 (2022). 37073218 10.1007/s42995-022-00130-5 PMC10077198

[79] Nisa, S., Wang, C., O’Leary, D. J., Powell, J. L. & Patel, N. Gaps in services and policy for arab older adults in Canada: A scoping review. *PLoS ONE* **19**(2), e0269536. 10.1371/journal.pone.0269536 (2024).

[80] Ajah, P. O. Growth Characteristics of the Monogonont Rotifer Asplanchna priodonta Gosse 1850 on Three Algae Species. *Turk. J. Fish. Aqua. Sci.* **8**, 275–282 (2008).

[81] Westerman, G. & Bonnet, D. The new elements of digital transformation. *MIT Sloan Manag. Rev.* **62**(2), 82–89 (2021).

[82] Muttaqin, I., Julyantoro, P. G. S. & Waskita Sari, A. H. Identification and predilection of ectoparasites of mangrove crabs (Scylla spp.) from the TAHURA Ngurah Rai mangrove ecosystem, Bali. *Curr. Trends Aquatic Sci.* **1**(1), 24–31. 10.2484/CTAS.2018.v01.i01.p04 (2018).

[83] Childers, R. K., Reno, P. W. & Olson, R. E. Prevalence and geographic range of *Nadelspora canceri* (Microspora) in Dungeness crab *Cancer magister*. *Dis. Aquat. Org.* **24**, 135–142. 10.3354/dao024135 (1996) (**(int-res.com)**).

[84] Davies, C. E. et al. Spatial and temporal disease dynamics of the parasite Hematodinium sp. in shore crabs, Carcinus maenas. *Parasit. Vectors* **12**(1), 472. 10.1186/s13071-019-3727 (2019). 31604479 10.1186/s13071-019-3727-x PMC6790014

[85] Shields, J. D. Hematodinium infections in crustaceans. *J. Invertebr. Pathol.* **166**, 107206. 10.1016/j.jip.2019.107206 (2019). 31152770 10.1016/j.jip.2019.107206

[86] Ruhay, A., İbrahim, C. The Investigation of Bacteria, Parasite and Fungi in Blue Crabs (Callinectessapidus, Rathbun 1896) Caught from Akyatan Lagoon in East Mediterranean Sea. 5th International Conference on Agriculture, Environment and Biological Sciences (ICAEBS-16) April 28–29, Pattaya Thailand (2016). 10.36630/jasft_20015

[87] Anderson RC. Nematoda parasites of vertebrates, their development and transmission.C.A.B international Wallingford; 99. Boschi EE 2000. Biodiversity of marine decapod brachyurans of the Americas. Journal of Crustacean Biology, 2: 337–342 )1992(

[88] Mehana, E.-S.E. et al. Biomonitoring of heavy metal pollution using acanthocephalans parasite in ecosystem: An updated overview. *Animals* **10**(5), 811. 10.3390/ani10050811 (2020). 32392878 10.3390/ani10050811 PMC7278602

[89] Sures, B. Host-parasite interactions in polluted environments. *J. Fish Biol.* **73**(9), 2133–2142. 10.1111/j.1095-8649.2008.02057 (2008).

[90] Grabner, D., Rothe, L. E. & Sures, B. Parasites and pollutants: Effects of multiple stressors on aquatic organisms. *Environ. Toxicol. Chem.* **42**(9), 1946–1959. 10.1002/etc.5689 (2023). 37283208 10.1002/etc.5689

[91] Fen, W. et al. Species-specific bioaccumulation of trace metals among fish species from Xincun Lagoon, South China Sea. *Sci. Rep.* **10**, 21800. 10.1038/s41598-020-77917-y (2020). 33311574 10.1038/s41598-020-77917-y PMC7732978

[92] Ortega-Jiménez, E., Cuesta, J. A., Laiz, I. & González-Ortegón, E. Diet of the invasive atlantic blue crab Callinectes sapidus Rathbun, 1896 (Decapoda, Portunidae) in the Guadalquivir estuary (Spain). *Estuar Coast* **47**, 1075–1085. 10.1080/095530099140311 (2024).

[93] Westerman, R. & Ahmed, M. *Oleksandr HolovachovSyst Parasitol* **99**, 83–101. 10.1007/s11230-021-10017-1(0123456789 (2022).

[94] Wu, Z., Wu, F. & Chen, X. Taxonomicandphylogenetic studies of two brackish *Pleuronema* species (Protista, Ciliophora,Scuticociliatia) from subtropical coastal waters of China, with report of a new species. *Microorganisms* **11**, 1422. 10.3390/microorganisms11061422 (2017). 10.3390/microorganisms11061422 PMC10304819 37374924

[95] Ziyaadini, M., Yousefiyanpour, Z., Ghasemzadeh, J. & Zahedi, M. Biota-sediment accumulation factor and concentration of heavy metals (Hg, Cd, As, Ni, Pb, and Cu) in sediments and tissues of Chiton lamyi (Mollusca: polyplacophora: Chitonidae) in Chabahar Bay Iran. *J. Fish. Sci.* **16**, 1123–1134 (2017).

